# Tysnd1 Deficiency in Mice Interferes with the Peroxisomal Localization of PTS2 Enzymes, Causing Lipid Metabolic Abnormalities and Male Infertility

**DOI:** 10.1371/journal.pgen.1003286

**Published:** 2013-02-14

**Authors:** Yumi Mizuno, Yuichi Ninomiya, Yutaka Nakachi, Mioko Iseki, Hiroyasu Iwasa, Masumi Akita, Tohru Tsukui, Nobuyuki Shimozawa, Chizuru Ito, Kiyotaka Toshimori, Megumi Nishimukai, Hiroshi Hara, Ryouta Maeba, Tomoki Okazaki, Ali Nasser Ali Alodaib, Mohammed Al Amoudi, Minnie Jacob, Fowzan S. Alkuraya, Yasushi Horai, Mitsuhiro Watanabe, Hiromi Motegi, Shigeharu Wakana, Tetsuo Noda, Igor V. Kurochkin, Yosuke Mizuno, Christian Schönbach, Yasushi Okazaki

**Affiliations:** 1 Division of Functional Genomics and Systems Medicine, Research Center for Genomic Medicine, Saitama Medical University, Hidaka-shi, Saitama, Japan; 2 Division of Translational Research, Research Center for Genomic Medicine, Saitama Medical University, Hidaka-shi, Saitama, Japan; 3 Division of Morphological Science, Biomedical Research Center, Saitama Medical University, Iruma-gun, Saitama, Japan; 4 Experimental Animal Laboratory, Research Center for Genomic Medicine, Saitama Medical University, Hidaka-shi, Saitama, Japan; 5 Division of Genomics Research, Life Science Research Center, Gifu University, Gifu, Japan; 6 Department of Anatomy and Developmental Biology, Graduate School of Medicine, Chiba University, Chiba, Japan; 7 Laboratory of Nutritional Biochemistry, Research Group of Food Science, Division of Applied Bioscience, Graduate School of Agriculture, Hokkaido University, Sapporo, Hokkaido, Japan; 8 Department of Biochemistry, Teikyo University School of Medicine, Itabashi-ku, Tokyo, Japan; 9 Developmental Genetics Department, Department of Genetics, King Faisal Specialist Hospital and Research Center, Riyadh, Kingdom of Saudi Arabia; 10 The National Newborn Screening Laboratory, Department of Genetics, King Faisal Specialist Hospital and Research Center, Riyadh, Kingdom of Saudi Arabia; 11 Department of Pediatrics, King Khalid University Hospital and College of Medicine, King Saud University, Riyadh, Kingdom of Saudi Arabia; 12 Department of Anatomy and Cell Biology, College of Medicine, Alfaisal University, Riyadh, Kingdom of Saudi Arabia; 13 Department of Internal Medicine, School of Medicine, Keio University, Shinjuku-ku, Tokyo, Japan; 14 Graduate School of Media and Governance, Keio University, Tokyo, Japan; 15 Faculty of Environment and Information Studies, Keio University, Tokyo, Japan; 16 Team for Advanced Development and Evaluation of Human Disease Models, Japan Mouse Clinic, BioResource Center (BRC), Tsukuba, Ibaraki, Japan; 17 The Japan Mouse Clinic, RIKEN BioResource Center (BRC), Tsukuba, Ibaraki, Japan; 18 The Cancer Institute of the Japanese Foundation for Cancer Research, Koto-ku, Tokyo, Japan; 19 Genome and Gene Expression Data Analysis Division, Bioinformatics Institute, A*STAR, Singapore, Republic of Singapore; 20 Division of Genomics and Genetics, School of Biological Sciences, Nanyang Technological University, Singapore, Republic of Singapore; Stanford University School of Medicine, United States of America

## Abstract

Peroxisomes are subcellular organelles involved in lipid metabolic processes, including those of very-long-chain fatty acids and branched-chain fatty acids, among others. Peroxisome matrix proteins are synthesized in the cytoplasm. Targeting signals (PTS or peroxisomal targeting signal) at the C-terminus (PTS1) or N-terminus (PTS2) of peroxisomal matrix proteins mediate their import into the organelle. In the case of PTS2-containing proteins, the PTS2 signal is cleaved from the protein when transported into peroxisomes. The functional mechanism of PTS2 processing, however, is poorly understood. Previously we identified Tysnd1 (Trypsin domain containing 1) and biochemically characterized it as a peroxisomal cysteine endopeptidase that directly processes PTS2-containing prethiolase Acaa1 and PTS1-containing Acox1, Hsd17b4, and ScpX. The latter three enzymes are crucial components of the very-long-chain fatty acids β-oxidation pathway. To clarify the *in vivo* functions and physiological role of Tysnd1, we analyzed the phenotype of *Tysnd1^−/−^* mice. Male *Tysnd1^−/−^* mice are infertile, and the epididymal sperms lack the acrosomal cap. These phenotypic features are most likely the result of changes in the molecular species composition of choline and ethanolamine plasmalogens. *Tysnd1^−/−^* mice also developed liver dysfunctions when the phytanic acid precursor phytol was orally administered. Phyh and Agps are known PTS2-containing proteins, but were identified as novel Tysnd1 substrates. Loss of *Tysnd1* interferes with the peroxisomal localization of Acaa1, Phyh, and Agps, which might cause the mild Zellweger syndrome spectrum-resembling phenotypes. Our data established that peroxisomal processing protease Tysnd1 is necessary to mediate the physiological functions of PTS2-containing substrates.

## Author Summary

Peroxisomes are subcellular organelles that are present in almost all eukaryotic cells. The syllables “per-oxi” reflect the oxidative functions of these single-membrane-bound organelles in various metabolic processes, including those of very-long-chain fatty acids and branched-chain fatty acids. In an earlier study we identified a protease named Tysnd1 that is specifically located in the peroxisomes and processes the enzymes catalyzing the peroxisomal β-oxidation of very-long-chain fatty acids. In this study, we identified two novel Tysnd1 substrates, Agps and Phyh, which are involved in plasmalogen synthesis and phytanic acid metabolism, respectively. To further investigate the *in vivo* function of Tysnd1, we analyzed *Tysnd1* knock-out mice. Mice that lack *Tysnd1* showed reduced peroxisomal β-oxidation activity and an altered plasmalogen composition, as well as an abnormal phytanic acid metabolism. Male infertility is one of the major phenotypic manifestations of *Tysnd1* deficiency. Our data support the idea that Tysnd1 affects the localization and activity of some of its substrates inside peroxisomes. Altogether, our *Tysnd1*-deficient mouse model expands the current peroxisome biology knowledge with regard to the molecular pathogenic mechanisms that may be relevant to some patients with Zellweger syndrome spectrum disorders.

## Introduction

Peroxisomes are subcellular organelles that are involved in the catabolism of very-long-chain fatty acids (VLCFAs), branched-chain fatty acids, D-amino acids, polyamines and the biosynthesis of bile acids [1]–[3]. Abnormalities of peroxisomal biogenesis or enzymes cause dysfunctions of the peroxisomal metabolism [3].

Clinically, peroxisomal disorders are divided into two large groups: Zellweger Syndrome spectrum (ZSS) and deficiency of peroxisomal enzymes [3]. ZSS is caused by defects of *PEX* (peroxisomal biogenesis factor) gene family members that interfere with or abrogate the biogenesis resulting in abnormally shaped peroxisomes or peroxisome deficiency [3]–[5]. In the case of ZSS peroxisome-targeted proteins are present in the cytosol, but most peroxisomal matrix proteins are not properly processed [6], [7].

ZSS includes neonatal adrenoleukodystrophy, infantile Refsum disease, rhizomelic chondrodysplasia punctata (RCDP) type 1 and Zellweger syndrome, the most severe form [3]. RCDP type 1 disease is caused by mutations in *PEX7* that interfere with its function as a receptor in targeting PTS2-containing proteins ACAA1 (acetyl-CoA acyltransferase 1), AGPS (alkylglycerone phosphate synthase) and PHYH (phytanoyl-CoA 2-hydroxylase) to the peroxisomes. The mutated *PEX7*-mediated effects result in the accumulation of VLCFAs, phytanic acid and a reduced plasmalogen synthesis [8].

Peroxisomal matrix proteins are imported from the cytoplasm into peroxisomes through PTSs [9], [10]. The majority of peroxisomal enzymes have a C-terminal PTS1 signal [SA]-K-L [11], that has been extended to a dodecamer motif [12]. PTS1 is recognized by the cytosolic soluble receptor Pex5p that carries the cargo to the peroxisomal membrane. A few peroxisomal proteins are targeted via the N-terminal PTS2 signal [RK]-[LVI]-[X5]-[HQ]-[LAF] [13] which is recognized by Pex7. Recently we identified the peroxisomal processing protease, Tysnd1 (trypsin domain containing 1) [14]. Tysnd1 is localized in peroxisomes and cleaves PTS2-containing prethiolase Acaa1, PTS1-containing proteins Acox1 (acyl-CoA oxidase 1, palmitoyl), Hsd17b4 (hydroxysteroid (17-beta) dehydrogenase 4) and ScpX [14], the longer mRNA product of gene *Scp2* (sterol carrier protein 2, liver) [15]. Scp2 lacks the thiolase domain-containing amino acids 1–404 of ScpX, but shares its C-terminal sequence (405–547) [15], [16]. Since Acox1, Hsd17b4 and ScpX are pivotal in the peroxisomal β-oxidation of VLCFAs, we created *Tysnd1^−/−^* mice to investigate the *in vivo* functions of Tysnd1, and to assess whether the phenotype would resemble any of the clinical features of human single peroxisomal enzyme deficiencies.

## Results

### Construction of *Tysnd1^−/−^* mice and phenotype screening

**Figure 1:**
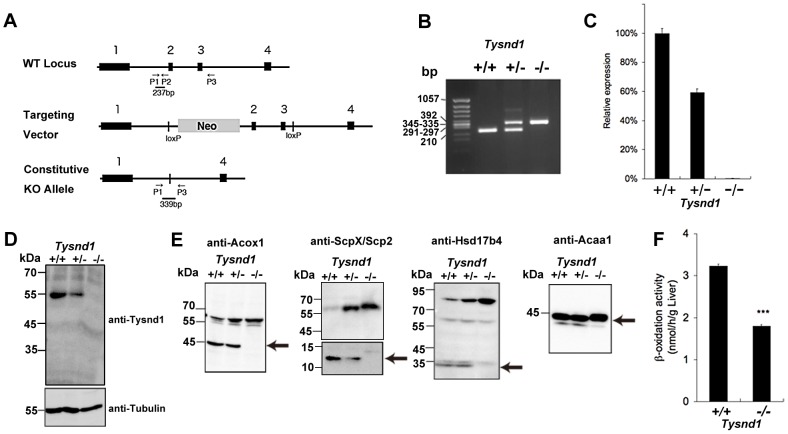
Generation of *Tysnd1* ^−/−^ mice. A. Map of *Tysnd1* ^−/−^ targeting constructs. P1, P2 and P3 indicate primers used for genotyping by PCR. B. Identification of genotyping by PCR. Wild type and *Tysnd1* ^−/−^ genotype were identified by 237 bp and 339 bp PCR products, respectively. C. Relative expression level of *Tysnd1* mRNA measured by quantitative real-time PCR in liver. D. Tysnd1 protein was absent in the liver of *Tysnd1* ^−/−^ mice as shown by Western blotting using anti-Tysnd1 antibody. E. The expression of known Tysnd1 substrates in the liver homogenate was detected by Western blotting using anti-Acox1, -ScpX/Scp2, -Hsd17b4 and -Acaa1 antibodies. Processed forms of Tysnd1 substrates were not detected in *Tysnd1* ^−/−^ mice. Arrows indicate the processed form of each enzyme. F. Peroxisomal β-oxidation activity was measured by [1-C^14^]lignoceric acids in 15 weeks old control diet-fed (CE2, Clea Japan) male mice liver homogenate. ***p<0.001. Each error bar represents the mean ± SE in *n* = 3.

*Tysnd1* was disrupted by targeted constitutive deletion of exons 2 and 3, encoding amino acids 392–496 of peptidase cysteine/serine, trypsin-like domain (333–537), using CRE/LoxP technology (Figure 1A and 1B). The *Tysnd1* ^−/−^ mice were obtained by crossing *Tysnd1* ^+/−^ mice. The ratio of homo- and heterozygote mice followed Mendel's law (1.08 vs. 2.18 for 285 homozygote mutants and 574 heterozygotes, compared with 1.0 for 263 wild-type mice). Tysnd1 mRNA and protein expression were completely disrupted in the *Tysnd1* ^−/−^ mice (Figure 1C and 1D). The phenotypes of female and male *Tysnd1* ^−/−^ mice of different litters were analyzed at 7–26 weeks of age using Japan Mouse Clinic (JMC) pipeline 1 [17], which includes modified-SmithKline Beecham, Harwell, Imperial College, Royal London Hospital phenotype assessment (modified-SHIRPA) [18] at eight weeks of age. *Tysnd1* ^−/−^ mice of both sexes did not display any anomalies with regard to body weight (eight weeks), body mass index (BMI, eight weeks), haematology (nine weeks), urine composition (ten weeks), anatomy (26 weeks), clinical blood biochemistry (eleven and 18 weeks), insulin tolerance (13 weeks), oral glucose tolerance (14 weeks), blood pressure (21 weeks), open field behaviour (seven weeks) and dual energy X-ray absorptiometry (22 weeks) compared with wild-type mice (data not shown). *Tysnd1* ^−/−^ male mice fed with high-fat diet between week 5 and 15 after birth did not display significant changes in body weight (Figure S1A), body length (Figure S1B) and BMI (Figure S1C) compared with age-matched male wild-type controls. Home cage activity [19] tested with 34 weeks old male mice was significantly reduced in male *Tysnd1* ^−/−^ mice (data not shown). The test was a component of a JMC-independent energy metabolism screen.

### *Tysnd1* ^−/−^ liver extracts contained only unprocessed substrates and showed reduced β-oxidation activity despite peroxisomal proliferation

Western blots of liver extracts prepared from 18 weeks old male *Tysnd1* ^−/−^ mice showed only increased amount of unprocessed Acaa1, Acox1, ScpX and Hsd17b4, whereas liver extracts from age- and gender-matched *Tysnd1* ^+/+^ and *Tysnd1* ^+/−^ mice contained both processed and unprocessed forms (Figure 1E). Electron microscopy (EM) image analysis (Figure S2A) of 29 weeks old male *Tysnd1* ^−/−^ liver sections revealed that the number of peroxisomes per counted area almost doubled compared with age- and gender-matched wild-type mice (Figure S2B). The slight increase of peroxisome size in *Tysnd1^−/−^* liver was statistically not significant (Figure S2C).

The peroxisomal β-oxidation activity measured by [1-^14^C]lignoceric acid oxidation in liver homogenates of 15 weeks old *Tysnd1* ^−/−^ male mice was reduced to approximately 60% of male wild-type mice of same litters (Figure 1F). The decreased β-oxidation activity did not affect blood serum VLCFA levels in adult (38–39 weeks) *Tysnd1* ^−/−^ male mice maintained on CE-2 diet compared with controls (data not shown).

### Male *Tysnd1* ^−/−^ mice are infertile and produce malformed sperms

**Figure 2:**
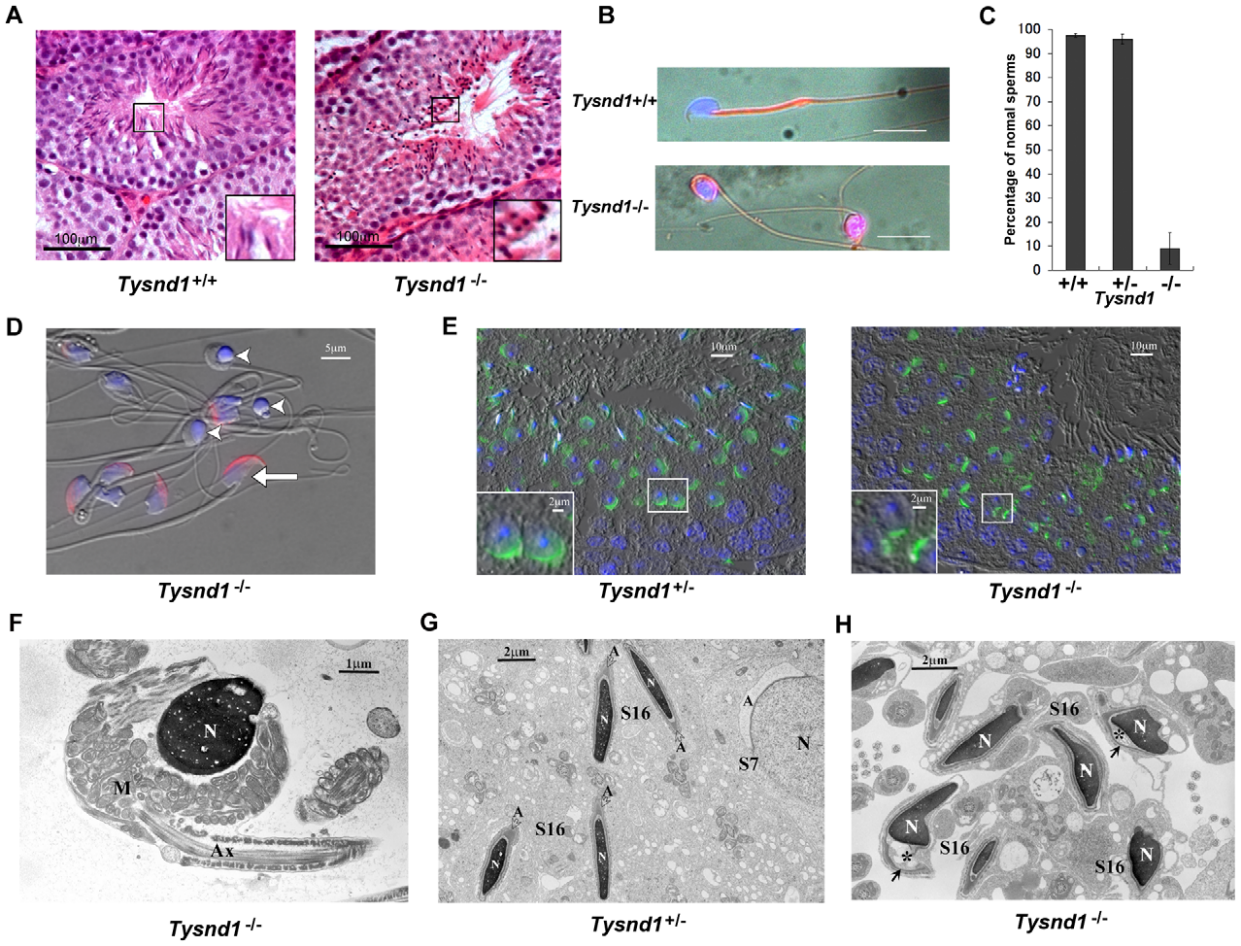
Male *Tysnd1* ^−/−^ mice are infertile. A. Semi-thin (8–10 micron) testes sections of 20 weeks old *Tysnd1* ^+/+^ and *Tysnd1^−/−^* mice were stained with hematoxylin-eosin. Abnormal, round sperm heads are visible in the seminiferous tubules of *Tysnd1* ^−/−^ mice. B. Abnormal morphology of *Tysnd1* ^−/−^ sperms. Epididymal sperms of 15 weeks-old *Tysnd1* ^+/+^ and *Tysnd1* ^−/−^ mice were stained with mitochondrial stains (MitoFluor Red) and nuclear staining (DAPI). Scale bar = 20 µm. C. Percentage of sperms showing normal morphology in *Tysnd1* ^+/+^ (n = 4), *Tysnd1* ^+/−^ (n = 4) and *Tysnd1* ^−/−^ (n = 5) mice. Each error bar represents the mean ± SE. D. Anti-MN9 antibody immunostaining and Hoechst nuclear staining of epididymal sperms isolated from a 20 weeks old *Tysnd1* ^−/−^ mouse (red: acrosome and blue: nucleus). Arrow heads and arrows indicate abnormal round-headed sperms and normal sperms, respectively. Scale bar = 5 µm. E. Acrosomes of a semi-thin testis section from a 10 weeks old *Tysnd1* ^−/−^ and a heterozygous control mouse were stained with PNA-FITC (green) and Hoechst nuclear stain (blue). F. EM image of a *Tysnd1* ^−/−^ caudal epididymal sperm. The round-headed sperm lacks the acrosome and shows an abnormal mitochondrial sheath (M) around the nucleus (N). Scale bar = 1 µm. G. EM image showing normal spermatogenesis in *Tysnd1* ^+/−^ male mice. S7: step 7 round spermatid; S16: step 16 elongated spermatid. Acrosomes (A) are normally formed. Scale bar = 2 µm. H. EM image of *Tysnd1* ^−/−^ elongated spermatid. S16: step 16 spermatid. In some spermatids the acrosome (*) is detached from the nucleus (N). Scale bar = 2 µm.

Repeated, independently conducted mating of wild-type female mice with male *Tysnd1* ^−/−^ mice produced no offspring (Table S1A). Mating pairs consisting of female *Tysnd1* ^−/−^ and wild-type or heterozygote males led to normal pregnancies and litters. The results lent support to our earlier formulated claim [14] that Tysnd1 may affect male fertility at the sperm level. The analysis of spermatogenesis revealed sperms with abnormally round-shaped heads in the seminiferous tubules of *Tysnd1* ^−/−^ mice (Figure 2A). Epididymal sperms of *Tysnd1* ^−/−^ mice showed coiled axonemes (Figure 2B), abnormal anterior acrosome lacking the acrosomal cap (Figure 2D). The defects were confirmed by staining with peanut agglutinin (PNA) lectin-conjugated FITC (Figure 2E) and by EM image analysis (Figure 2F and 2G). Since the anterior acrosome anomaly may affect the acrosome reaction we conducted sperm penetration assays using *in vitro* fertilization under intact cumulus mass and zonae pellucidae-free conditions (Table S1B). When the cumulus mass was intact, fertilization by *Tysnd1* ^−/−^ sperms was significantly reduced.

### Several plasmalogen species are reduced in *Tysnd1* ^−/−^ mice

**Figure 3:**
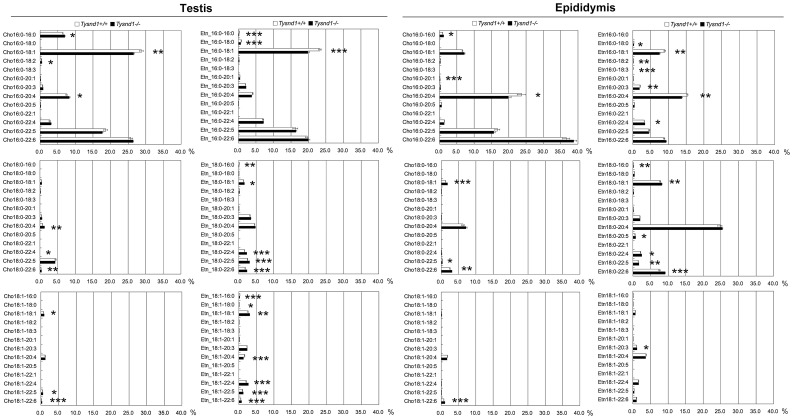
The composition of plasmalogen molecular species (%) in testes and epididymides. Plasmalogen levels of whole testes and epididymides of *Tysnd1* ^+/+^ (white bars) and *Tysnd1^−/−^* (black bars) mice were measured by LC/ESI-MS/MS. Each error bar represents the mean ± SE in n = 5. * *p*<0.05, **p<0.01 and *** *p*<0.001.

Phospholipids are primary components of cellular membranes. Since the deciduation of sperm acrosomes occurred only in *Tysnd1* ^−/−^ mice (Figure 2D and 2E), we hypothesized that alterations in the phospholipid composition causes the fragility of acrosomal membrane. Plasmalogens are major components of the acrosomal membrane [20]. The first two steps of plasmalogen synthesis, which are catalyzed by Gnpat (glyceronephosphate O-acyltransferase) and Agps occur in the peroxisomes [21]. We assessed the effect of Tysnd1 loss on plasmalogens by measuring the ethanolamin or choline plasmalogen species composition of the vinyl ether bound fatty alcohol at *sn*-1 and the ester bound fatty acids at *sn*-2 position of the glycerol backbone using whole testes and epididymides extracts of ten to eleven weeks old *Tysnd1* ^−/−^ and wild-type males. Although we did not find significant differences in total plasmalogen levels between *Tysnd1* ^−/−^ and wild-type mice (Figure S3), we detected differences in the ratio and composition of certain plasmalogens. In testes (Figure 3A and 3B) palmitic acid-oleic acid (16∶0–18∶1) prevailed among both choline and ethanolamine plasmalogens with 16∶0–18∶1 slightly decreased in *Tysnd1* ^−/−^ mice. Choline- and ethanolamine-type plasmalogens were reduced 8.0% and 14.7%, respectively. Among *Tysnd1* ^−/−^ epididymal ethanolamine plasmalogens we observed a 16.7% decrease in 16∶0–18∶1 and a 10.5% decrease in 16∶0–20∶4 palmitic acid-arachidonic acids compared with wild-type controls (Figure 3). Epididymal choline plasmalogens were only reduced at 16∶0–20∶4 levels (16.5% decrease) (Figure 3). In contrast, plasmalogen containing DPA (docosapentanoic acid; 22∶5) or DHA (docosahexaenoic acid; 22∶6) at *sn*-2 position were slightly higher in testes and epididymides of *Tysnd1* ^−/−^ mice than in wild-type mice (Figure 3).

### Phytanic acid and VLCFA metabolism is reduced in *Tysnd1* ^−/−^ mice

**Figure 4:**
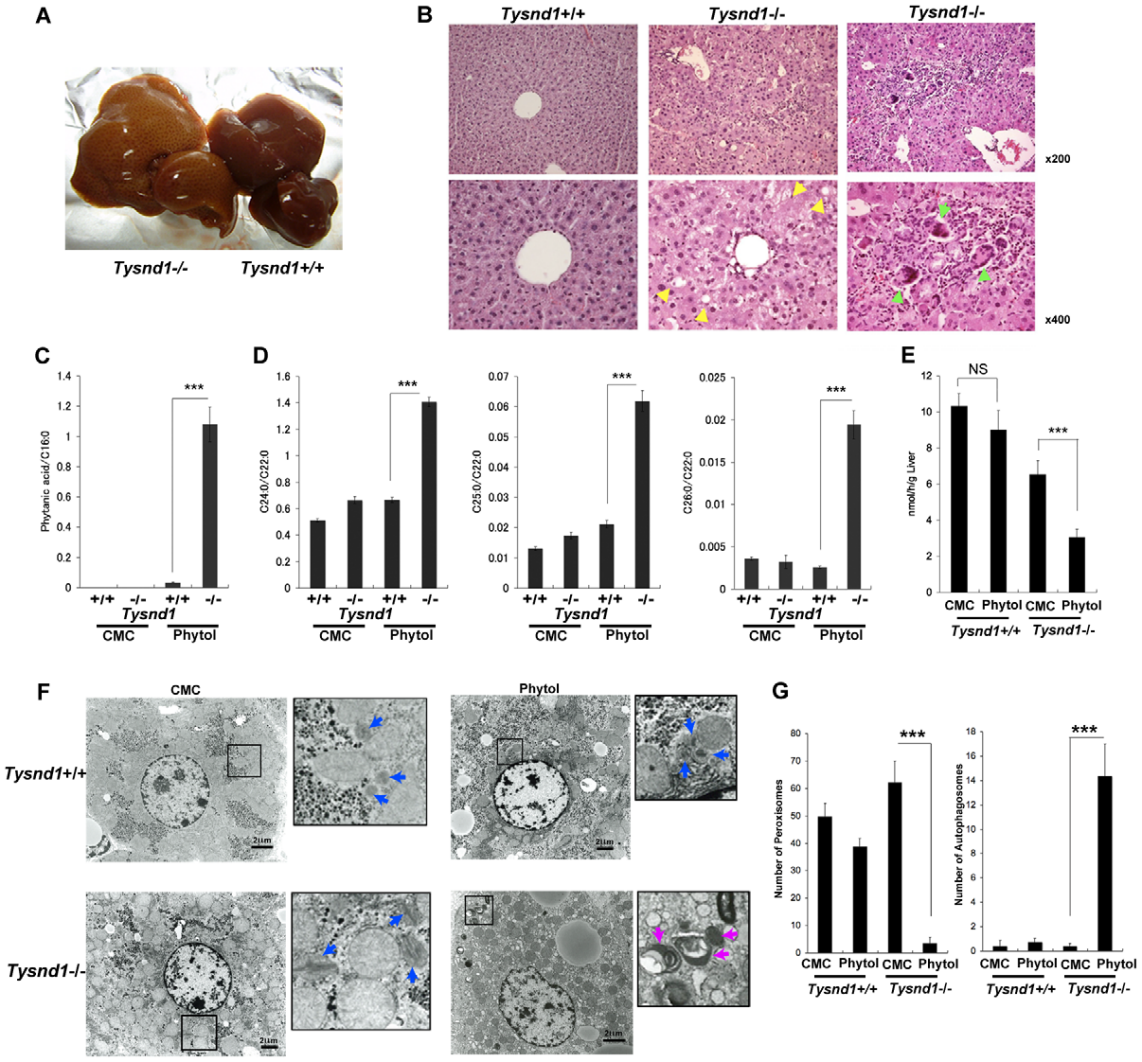
Ten-week-old male *Tysnd1* ^−/−^ mice accumulate phytanic acid. A. Livers after 13 days of phytol feeding. Left: enlarged, beige-coloured liver of phytol-fed *Tysnd1* ^−/−^ mouse. Right: liver of phytol-fed *Tysnd1* ^+/+^ mouse. B. Hematoxylin-eosin stained semi-thin liver sections. Lipid droplets are indicated by yellow arrow heads. Green arrow heads indicate giant cells. C. Blood serum-derived phytanic acid measurement by GC/MS normalized for C16∶0 (hexadecanoic acid) content. Each error bar represents the mean ± SE in n = 4–9. ***p<0.001. D. Measurement of blood serum C24∶0 (tetracosanoic acid), C25∶0 (pentacosanoic acid) and C26∶0 (hexacosanoic acid) VLCFAs by GC/MS normalized by C22∶0 (docosanoic acid) content. Each error bars: mean ± SE in n = 4–9. ***p<0.001. E. Measurement of liver peroxisomal β-oxidation activity with or without orally administered 0.5% phytol in carboxyl methyl cellulose (CMC). Each error bars: mean ± SE in *n* = 3–6. NS: not significant. ***p<0.001. F. EM image of liver sections with or without orally administered phytol in CMC. The blue and purple arrow heads indicate peroxisomes and autophagosomes, respectively.Bar: 2 µm. G. Numbers of peroxisomes and autophagosomes counted within same field areas of EM images. Error bars: mean ± SE in n = 5–8. ***p<0.001.

The Tysnd1 substrates Hsd17b4 and ScpX catalyze the oxidation of branched-chain fatty acids. In human, defects of *Hsd17b4* cause various neurological abnormalities [22], limb abduction and hypotonia. In *Scp2*- and *Hsd17b4*-deficient mice phytanic acid accumulates in the liver when its precursor phytol is orally administered [23], [24]. We tested whether *Tysnd1* ^−/−^ mice would show a similar abnormal biochemical profile. After determining phytanic acid levels in the blood serum of 38–39 weeks old male *Tysnd1* ^−/−^ mice we found that phytanic acid levels were significantly higher in *Tysnd1* ^−/−^ mice than in age-matched male wild-type mice (Figure S4A). In the absence of visible macroscopic abnormalities, we performed a phytol (15 mg/day) overloading experiment over a period of 13–14 days with eight weeks old mice. All female *Tysnd1* ^−/−^ mice on the phytol-containing diet died after one week and were not further analyzed. Male *Tysnd1* ^−/−^ mice lost approximately 20% of their body weight compared with 5% in wild-type mice (Figure S4B and S4C). Phytol-fed male *Tysnd1* ^−/−^ mice had beige livers (Figure 4A) that contained a two times greater amount of total fat (Figure S4D) and triglycerides (Figure S4E) than controls, indicating the onset of a fatty liver phenotype. The liver parenchyma of *Tysnd1* ^−/−^ mice appeared inflamed and was infiltrated by giant cells (Figure 4B). Phytanic acid accumulated in the sera of phytol-administered male *Tysnd1* ^−/−^ mice more than 100-fold compared with controls (Figure 4C). Plasma pristanic acid, a metabolite of phytanic acid was elevated in ten weeks old male *Tysnd1* ^−/−^ mice that were fed for ten days with phytol, but statistically not significant when compared to wild-type mice (Figure S4G).

The blood serum of phytol-fed *Tysnd1^−/−^* mice showed a significant accumulation of VLCFAs (Figure 4D) compared with wild-type mice and controls on a carboxy methyl cellulose (CMC) diet. After phytol feeding, the ratios of tetracosanoic acid (C24∶0) docosanoic acid (C22∶0), pentacosanoic acid (C25∶0) and hexacosanoic acid (C26∶0) to docosanoic acid (C22∶0) increased between *Tysnd1^+/+^* mice from 0.66 for C24∶0, 0.017 for C25∶0 and 0.0011 for C26∶0 to 1.41, 0.062 and 0.0034 in *Tysnd1^−/−^* mice, respectively. The reasons for the somewhat peculiar higher C25∶0/C22∶0 than C26∶0/C22∶0 ratios in both, wild-type and *Tysnd1^−/−^* mice are unknown.

### Macropexophagy and liver dysfunction of phytol-administered male *Tysnd1^−/−^* mice

The increase in VLCFAs after phytol feeding of *Tysnd1^−/−^* mice was accompanied by a significantly reduced liver peroxisomal β-oxidation rate compared with CMC-fed controls and phytol-fed wild-type mice (Figure 4E). Liver mitochondrial β-oxidation was not affected after phytol feeding of *Tysnd1^−/−^* mice (Figure S4F). EM images of liver tissue sections of CMC-fed *Tysnd1* ^−/−^ mice showed enlarged peroxisomes (Figure 4F) compared with wild-type mice. In addition, we observed in phytol-fed *Tysnd1^−/−^* mice a significant decrease in the number of liver peroxisomes and an increase in autophagosomes (Figure 4F and 4G). Since the mitochondria appeared intact (Figure 4F; Figure S4F), the autophagosomes are likely to be of peroxisomal origin and of the macropexophagy type. The observed changes indicate that phytol administration to *Tysnd1^−/−^* mice causes substantial peroxisomal dysfunctions.

Clinical blood biochemical analyses of *Tysnd1^−/−^* mice administered with phytol showed significantly elevated levels of lactate dehydrogenase (LDH), aspartate transaminase (AST) and alanine aminotransferase (ALT) that are indicative of liver damage (Table S2).

### Tysnd1 processes PTS2-containing proteins Agps and Phyh

**Figure 5:**
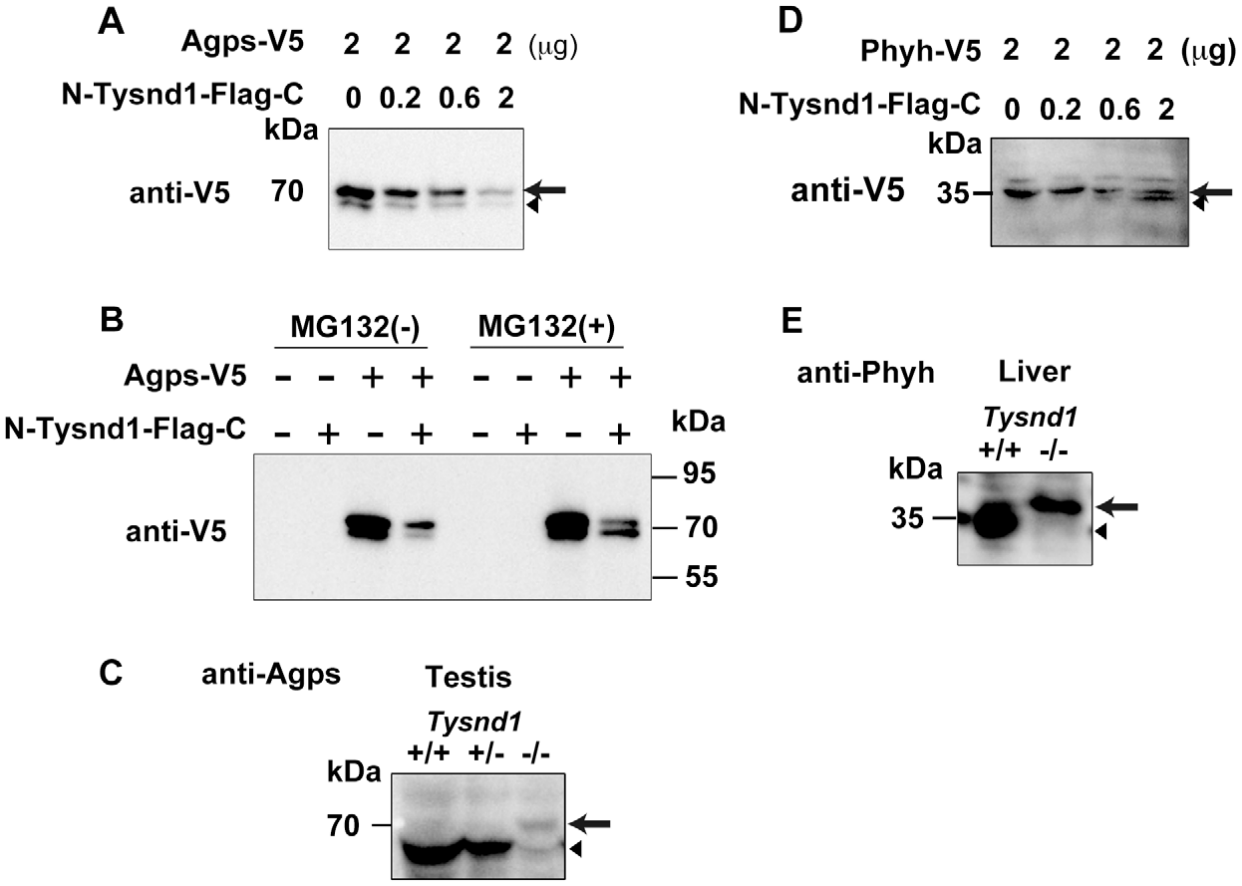
Tysnd1 processes Agps and Phyh *in vitro*. A. COS7 cells were transiently co-transfected with Agps-V5 and Tysnd1 expression plasmids. With increasing amounts of Tysnd1 unprocessed Agps-V5 decreased (arrow). Processed Agps-V5 is indicated by an arrowhead B. Agps processing by Tysnd1 is specific and was affected by MG132 proteasome inhibitor. C. Western blot of testes extract using anti-Agps antibody shows unprocessed (arrow) and processed (arrowhead) forms of Agps. D. COS7 cells were transiently co-transfected with Phyh-V5 and Tysnd1 expression plasmids. With increasing amounts of Tysnd1, unprocessed Phyh-V5 decreased (arrow) and processed Phyh-V5 increased (arrowhead). E. Western blot of liver extract using anti-Phyh antibody shows unprocessed (arrow) and processed (arrowhead) forms of Phyh.

Gnpat, Agps, Far1 (fatty acyl CoA reductase 1) and Far2 (fatty acyl CoA reductase 2) are enzymes involved in plasmalogen synthesis. Phytanic acid oxidation depends on Phyh, whereas Amacr (alpha-methylacyl-CoA racemase) is involved in the β-oxidation of pristanic acid [25]. We co-transfected COS-7 cells with Agps and Tysnd1 or Phyh and Tysnd1 while increasing the amount of Tysnd1 to evaluate its substrate processing. The unprocessed forms of Agps and Phyh decreased (Figure 5A, 5D) in proportion to the increase of Tysnd1. Since the amount of processed Phyh increased, Tysnd1 seems to process Phyh directly. The amount of processed Agps increased only in presence of proteasome inhibitor MG132 indicating possible degradation via the ubiquitin-proteasome pathway (Figure 5A, 5B). Western blot analyses of testes and liver extracts support *in vivo* processing of Agps and Phyh by Tysnd1. The processed forms of Agps and Phyh were present in wild-type and heterozygous mice, but absent in the extracts of *Tysnd1^−/−^* mice (Figure 5C, 5E).

Furthermore we tested whether Tysnd1 can process Gnpat, Amacr, Far1 and Far2. Peroxisomal membrane-bound Far1 and Far2 are involved in plasmalogen synthesis, but localized to peroxisomes in an apparently PTS1/PTS2-independent manner [26]. Tysnd1 co-transfection experiments of COS-7 cells with Gnpat, Amacr, Far1 and Far2 demonstrated that the amount of unprocessed Gnpat and Far2 decreased (Figure S5A, S5C). The *in vivo* evidence of Gnpat and Far2 as Tysnd1 substrates remained ambiguous due to faint Western blot signals (data not shown). Far1 and Amacr were not affected by Tysnd1 co-transfection, implying that these enzymes are not substrates of Tysnd1 (Figure S5B, S5D).

### Tysnd1 deficiency interferes with the peroxisomal localization of PTS2-containing proteins

**Figure 6:**
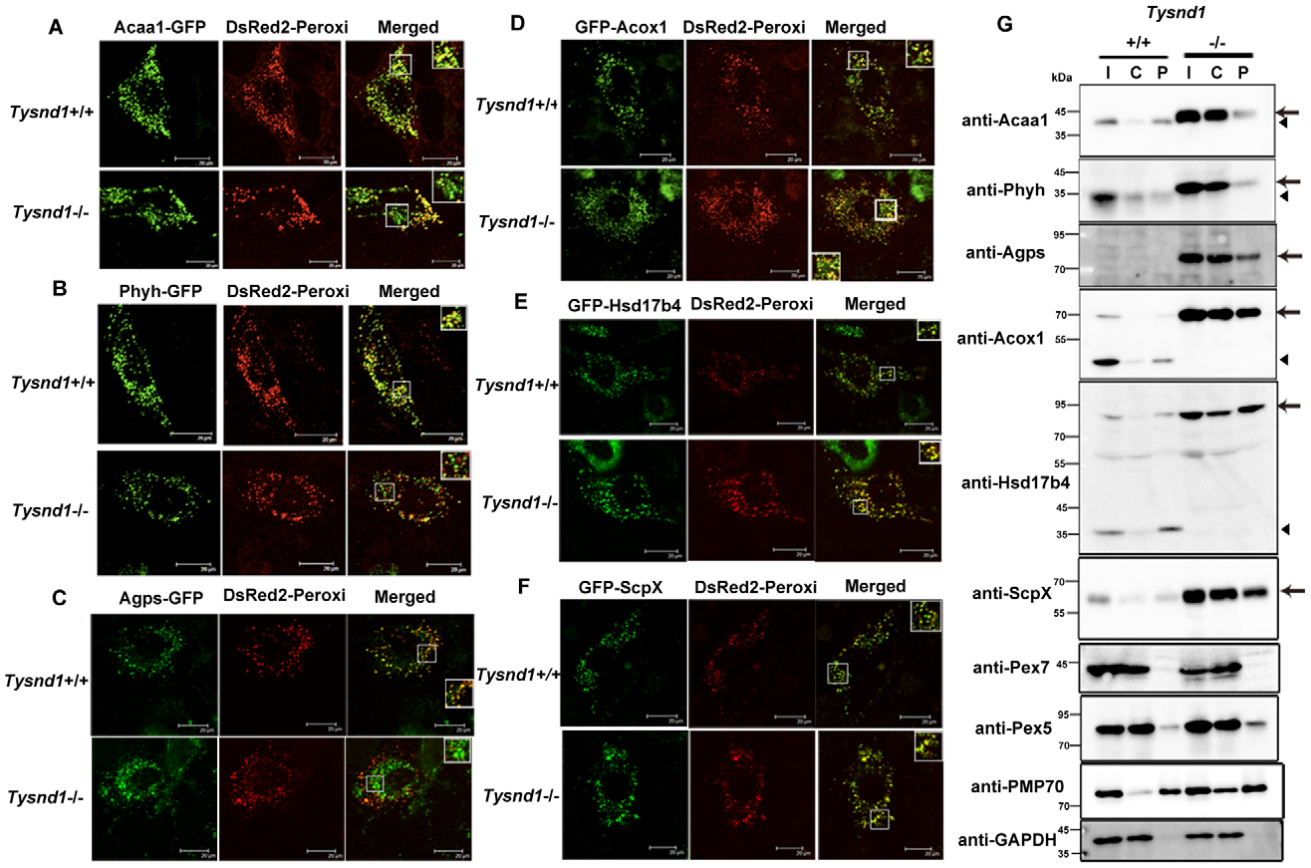
PTS2-containing proteins poorly localized to peroxisomes in *Tysnd1^−/−^* primary hepatocytes. Confocal laser-scanning microscopy of *Tysnd1* ^−/−^ and *Tysnd1^+/+^* primary hepatocytes co-transfected with Tysnd1 GFP-substrate constructs (green) and peroxisomal marker DsRed2-Peroxi (red). Small boxes indicate image sections that were magnified 1.5 times and shown in large boxes. A. Acaa1-GFP (PTS2). B. Phyh-GFP (PTS2). C. Agps-GFP (PTS2). D. GFP-Acox1 (PTS1). E. GFP-Hsd17b4 (PTS1). F. GFP-ScpX (PTS1). G. Western blot of fractionated liver protein extracts of *Tysnd1* ^−/−^ and wild-type. I: input (post-nuclear fraction), C: cytosol-rich fraction, P: peroxisome-rich fraction. Arrow: unprocessed forms of Tysnd1 substrates, A: Arrowhead: processed form of Tysnd1 substrates. Pex5 and Pex7 are the PTS1 and PTS2 receptors, respectively. Pmp70 is a peroxisomal membrane marker and Gapdh a marker for post-nuclear and cytosol-rich fractions.

Assuming that Tysnd1 processing of peroxisomal proteins is essential for the their localization to peroxisomes, we assayed the localization of each Tysnd1 substrate, Acaa1, Phyh, Agps, Acox1, Hsd17b4, and ScpX by co-transfecting expression vector constructs for the substrate-GFP fusion proteins with peroxisomal location marker DsRed2-Peroxi (PTS1) into primary hepatocytes of six weeks old *Tysnd1^−/−^* and wild-type male mice. The subcellular localization was assessed by confocal laser-scanning microscopy 27–28 hours after transfection (Figure 6A–6F). In wild-type mice hepatocytes all six GFP fusion proteins unequivocally co-localized with DsRed2-Peroxi, indicating their peroxisomal localization (Figure 6A–6F; Figure S6A–S6F). In contrast, in *Tysnd1^−/−^* hepatocytes PTS2- containing GFP fusion proteins Acaa1, Phyh and Agps (Figure 6A–6C; Figure S6A–S6C) co-localized to a noticeably lesser degree with DsRed2-Peroxi than PTS1-containing GFP fusion proteins Acox1, Hsd17b4 and ScpX (Figure 6D–6F; Figure S6D–S6F), which showed mainly peroxisomal localization. In *Tysnd1^−/−^* hepatocytes, successfully transfected with Acaa1-GFP, some of it was observed to co-localize in part with peroxisomes and some remained outside the peroxisomes (Figure 6A; Figure S6A). Most of Phyh-GFP appeared as punctuated structures with some co-localized with DsRed2-Peroxi (Figure 6B; Figure S6B). Almost all Agps-GFP did not co-localize with DsRed2-Peroxi, indicating mostly non-peroxisomal localization (Figure 6C; Figure S6C).

In a control experiment we also tested the suitability of DsRed2-Peroxi (PTS1) as peroxisomal co-localization marker in *Tysnd1^−/−^* hepatocytes by comparing its co-localization with anti-Pmp70 Alexa Fluor 488, an antibody against peroxisomal membrane marker Abcd3 (ATP-binding cassette, sub-family D (ALD), member 3) also called Pmp70. Both markers co-localized with peroxisomes, confirming that the peroxisomal localization properties of DsRed2-Peroxi itself were not affected in *Tysnd1^−/−^* hepatocytes (Figure S7A). Since co-transfection of DsRed2-Peroxi with Tysnd1 substrates expressed as GFP fusion proteins might strain the peroxisomal protein import capacity we evaluated the co-localization of singly transfected Acaa1-GFP, Phyh-GFP and GFP-Hsd17b4 with anti-Pmp70 Alexa Fluor 568. Under singly-transfection conditions, Acaa1-GFP (Figure S7B) and Phyh-GFP (Figure S7C) co-localized with peroxisome marker anti-Pmp70 in both *Tysnd1^−/−^* and *Tysnd1^+/+^* hepatocytes. Similarly for GFP-Hsd17b4, we did not observe any differences in the peroxisomal co-localization with anti-Pmp70 between *Tysnd1^−/−^* and *Tysnd1^+/+^* hepatocytes (Figure S7D). Altogether, the localization of PTS2 matrix proteins in *Tysnd1^−/−^* hepatocytes was only affected when the PTS1-containing DsRed2-Peroxi marker was co-transfected.

Western blot analysis of the liver subcellular fractions showed that a considerable amount of unprocessed Acaa1, Phyh, and Agps were localized to the cytosol-enriched fraction of *Tysnd1* ^−/−^ liver. In wild-type mice liver extracts Acaa1 and Phyh were almost exclusively detected in the peroxisome-enriched fraction, and Agps was not detectable in wild-type mice livers (Figure 6G). The results of Agps processing by Tysnd1 in COS7 cells supplemented with MG132 proteasome inhibitor indicate that the processed form of Agps is prone to degradation (Figure 5B). The unprocessed forms of PTS1-containing proteins Acox1, Hsd17b4, and ScpX were enriched in the liver peroxisome- and cytosol-enriched fractions of *Tysnd1* ^−/−^ mice compared with wild-type mice (Figure 6G). The results are consistent with the increased amount of unprocessed Acaa1, Acox1, ScpX, and Hsd17b4 observed on Western blots of liver extracts (Figure 1E) and the expression pattern of peroxisomal membrane marker Pmp70 in *Tysnd1* ^−/−^ (Figure 6G). Pex5, the peroxisomal PTS1 receptor, was present in the cytosol- and peroxisome-enriched fractions of both *Tysnd1* ^+/+^ and *Tysnd1* ^−/−^ liver, with the majority located in the cytosolic fraction (Figure 6G). Pex7, the PTS2 receptor was barely detected in the peroxisomal fractions of *Tysnd1* ^+/+^ and *Tysnd1* ^−/−^ liver.

We interpret the partial peroxisomal localization of PTS1- and PTS2-containing proteins observed in the fractionation experiment (Figure 6G) as the effect of an overall accumulation of peroxisomal proteins in *Tysnd1^−/−^* primary hepatocytes. Loss of Tysnd1 might mediate the overload of the peroxisomal targeting signal receptors. To test this hypothesis, we co-transfected *Tysnd1^+/+^* and *Tysnd1^−/−^* hepatocytes with Acaa1-GFP (4 µg) and HA-Acox1 (1 µg and 4 µg), and compared the co-localization of Acaa1-GFP (PTS2) with anti-Pmp70 Alexa Fluor 568 to that in singly-transfected hepatocytes. In *Tysnd1^−/−^* hepatocytes Acaa1-GFP localized in part with peroxisomes and in part with a different cellular compartment (Figure S7E). In contrast, even after co-transfection of *Tysnd1^+/+^* hepatocytes with 1 µg HA-Acox1 (Figure S7E) or 4 µg HA-Acox1 (data not shown), Acaa1-GFP still co-localized with Pmp70 Alexa Fluor 568 in peroxisomes, indirectly lending support to our hypothesis.

## Discussion

Tysnd1 processes both PTS1-targeted (Acox1, Hsd17b4, and ScpX) and PTS2-targeted (Agps, Phyh, and Acaa1) enzymes that are involved in peroxisomal β-oxidation of VLCFAs, phytanic acid metabolism, and plasmalogen synthesis. In Tysnd1-deficient mice limited peroxisomal targeting and accumulation of unprocessed substrates reduced the metabolic activities of the aforementioned three pathways. As shown in Figure 1E and Figure 6G, the amount of the examined peroxisomal matrix proteins and total Pmp70 was elevated in *Tysnd1* ^−/−^ mice, indicating spontaneous peroxisome proliferation (Figure 6A). We interpret the unexpectedly strong Pmp70 signal in the cytosol-enriched fraction (Figure 6G) of *Tysnd1^−/−^* liver extract as possible interference with the chaperoning function of Pex19, which binds co-translationally to newly synthesized Pmp70 and is docked by Pex3 to the peroxisomal membrane [27], [28]. Since Pmp70 was reported to aggregate and degrade in absence of Pex19 [27], we speculate that Tysnd1 deficiency might somehow indirectly interfere with the insertion of Pmp70 into the peroxisomal membrane via Pex19-Pex3 docking, leaving soluble Pmp70 in the cytoplasm. However, without further investigation we cannot exclude other mechanisms that account for the presence of Pmp70 in the cytosol-enriched fraction.

EM image analysis of liver peroxisomes in *Tysnd1* ^−/−^ mice showed a significant increase in their number (Figure S2B) together with a slight enlargement (Figure S2C). The proliferation of peroxisomes in *Tysnd1* ^−/−^ mice seems to be the result of compensatory changes caused by their impaired peroxisomal functions. The resulting phenotype of *Tysnd1* ^−/−^ mice resembles biochemically mild variants of RCDP type 1 disease (RCDP1) with somewhat decreased plasmalogen and increased phytanic acid levels. RCDP1 is caused by nonsense mutations in PEX7 which specifically prevent the import of PTS2-containing proteins PHYH, ACAA1, and AGPS [29]–[31] into peroxisomes. *Pex7* ^−/−^ mice, a model for RCDP1 [8] show reduced plasmalogen synthesis. In *Pex7* ^−/−^ mice the α-oxidation of phytanic acid is impaired, resulting in low phytanic acid levels under normal diet conditions, but a significant accumulation after oral phytol administration. At birth only 50% of pups were alive, and the surviving male mice developed testicular atrophy with infertility, dwarfism by delayed endochondral ossification and eye cataracts in adults. In contrast, *Tysnd1* ^−/−^ mice displayed a rather mild phenotype with most pups reaching adulthood without dwarfism and normal eyes until we stopped maintaining and monitoring the mice at one year of age (data not shown). The underlying phenotypic differences are attributed to the abrogation of PTS2-containing peroxisomal protein import in *Pex7* ^−/−^ mice versus diminished import with inadequate peroxisomal localization in *Tysnd1* ^−/−^ mice. Similar findings were reported for mice deficient in Gnpat, which is also involved in plasmalogen synthesis. Male *Gnpat^−/−^* mice are aspermic and infertile [32], whereas infertile *Tysnd1* ^−/−^ mice produce malformed sperms.

Biochemically, classical RCDP1 patients show strongly reduced plasmalogen, elevated phytanic acid and low pristanic acid levels [33]. Clinical symptoms include severe growth and mental retardation, congenital cataracts, chondrodysplasia and rhizomelia. A biochemical and neurological study of eleven patients diagnosed with RCDP1 included three female patients who were clinically diagnosed with a mild form of RCDP1 [34]. One of the patients who displayed autistic behaviour patterns and developed epilepsy at age 21 had only weakly elevated phytanic acid and reduced plasmalogen levels [34]. It is therefore possible that TYSND1 deficiency in human might cause phenotypes that are clinically diagnosed as a mild RCDP1 variant accompanied by male infertility.

Repeated unsuccessful mating of male and female *Tysnd1* ^−/−^ mice helped us to discover the abnormal sperms, which prompted us to conduct a plasmalogen analysis. Plasmalogens are phospholipids that are enriched in myelin [21], [35], testes and spermatozoa membranes [20], [36] where they are involved in anti-apoptotic functions [37] and spermatogenesis [38]. Developing gametes mature in the epididymis where the remodelling of ether lipids that constitute the sperm cell membrane occurs [39]. In *Tysnd1* ^−/−^ hepatocytes Agps, a rate-limiting enzyme [40] in the peroxisomal steps of plasmalogen synthesis showed almost no peroxisomal co-localization with DsRed2-Peroxi when transfected as GFP construct (Figure 6C). The changes in the composition of choline- and ethanolamine-type components of epididymal plasmalogens (Figure 3) most likely result in fragile sperm cell membranes and missing or defect acrosomes. The acrosome contains digestive enzymes that break down the outer ovum membrane, zona pellucida. Therefore, the acrosome-deficient sperms of *Tysnd1^−/−^* mice are unable to penetrate through the cumulus to fertilise the egg.

Plasmalogens are also involved in neurodegenerative diseases [41]. In Alzheimer model mice levels of Agps protein and plasmalogen synthesis are reduced [42]. Considering that phytanic acid accumulates in the plasma, liver and brain of phytol-fed Pex7-deficient mice [8] and RCDP patients [29], [43] the significantly lower home cage activity (data not shown) of male *Tysnd1^−/−^* mice at 34 weeks of age might be an indicator of behavioural anomalies due to neuronal changes mediated by reduced plasmalogen and elevated phytanic acid levels. Neurological and biochemical analyses of the central and peripheral nervous system are on-going and will be reported elsewhere.

Phytanic acid, a natural agonist of PPARα induces peroxisome proliferation and hypertrophy [44]. Since blood serum levels of phytanic acid in male *Tysnd1^−/−^* mice fed with normal diet (CLEA Japan) were elevated (Figure S4A), the metabolic tolerance to phytol, a precursor of phytanic acid was tested in a phytol feeding experiment. Serum phytanic acid levels of male *Tysdn1^−/−^* mice increased 100-fold compared with phytol-administered male wild-type mice.

The phytol-intolerant phenotype and hepatic lipidosis seen in*Tysnd1^−/−^* mice is similar to *Phyh^−/−^* mice. However male *Phyh^−/−^* mice are born without any abnormalities, and they are fertile in contrast to *Tysnd1^−/−^* mice. Ferdinandusse *et al.* [45] showed that phytol-fed *Phyh^−/−^* mice developed ataxia due to accumulation of phytanic acid in the cerebellum. Although *Tysnd1^−/−^* mice received twice the amount of phytol as the *Phyh^−/−^* mice [45] we did not observe ataxia, indicating only a mild impairment of phytanic acid metabolism in *Tysnd1^−/−^* mice. The study of an *E. coli*-expressed PHYH with a mutation in the N-terminal PTS2 region demonstrated that the unprocessed form of PHYH is active, but may affect *in vivo* its solubility and/or transport into peroxisomes [46]. We showed that unprocessed Phyh had a much stronger signal in the cytosol-enriched fraction of *Tysnd1^−/−^* mice (Figure 6G) than in the peroxisomal fraction. If unprocessed Phyh is active *in vivo*, it is probably the diminished amount of unprocessed Phyh inside peroxisomes that reduces phytanic acid metabolism in *Tysnd1^−/−^* mice.

The peroxisomes of phytol-administered *Tysnd1^−/−^* mice showed a concomitant decrease in their number and an increase in their size. Probably, the extreme Pparα-mediated hypertrophy induced autophagy of peroxisomes (Figure 4F, 4G), also termed pexophagy [47], [48]. The accumulation of unprocessed peroxisome-targeted proteins Acox1, Hsd17b4, ScpX and Acaa1 (Figure 1E) in male phytol-administered *Tysnd1^−/−^* mice indicates the induction of a compensatory mechanism that counteracts the Tysnd1 deficiency-mediated reduced metabolic functions of peroxisomes and inflammatory liver changes.

The inflammatory liver changes seen in *Tysnd1^−/−^* mice administered with phytol resembled the liver phenotypes of *Scp2*-deficient mice [23]. Two out of nine male mice suddenly died at days 12 and 13 after phytol administration. Possibly, atrioventricular changes induced by high phytanic acid levels led to cardiac arrest as reported for *Scp2*-knockout mice [49]. All female *Tysnd1^−/−^* mice administered with phytol-containing diet died during the experiment. BALB/c and C57BL/6J females are known to have low amounts of liver ScpX, which catalyses the thiolytic cleavage of branched-chain 3-ketopristanoyl-CoA during the degradation of pristanic acid [50]. Tysnd1 loss of function in female mice seems to exacerbate the effect of reduced amounts of ScpX and the toxicity of accumulating phytanic acid. Although plasma pristanic acid levels of phytol-fed *Tysnd1^−/−^* mice were statistically not significantly (p = 0.105) elevated (Figure S4G), the significant increase in phytanic acid (Figure 4C) suggests that the loss of Tysnd1 affects Phyh, the primary peroxisomal α-oxidation enzyme and subsequent enzymes in the hierarchy of β-oxidation reactions as evidenced by the reduced peroxisomal β-oxidation rate in liver (Figure 4E).

An *in vitro* study showed that there are no differences in the enzymatic activity of processed and unprocessed forms of human AGPS [51]. Since peroxisomes lack DNA and protein synthesis capabilities, all peroxisomal proteins are synthesized in the cytosolic compartment and post-translationally sorted to the peroxisome. Insufficient cleavage of substrates interfered with their peroxisomal localization as observed in primary hepatocytes of *Tysnd1^−/−^* mice (Figure 6A–6C; Figure S6A–S6C, Figure S7E). Reduced peroxisomal targeting neither occurred in wild-type hepatocytes (Figure 6A–6F; Figure S7B–S7D) nor when peroxisomal protein Acox1 was co-expressed (Figure S7E) or overexpressed (data not shown), indicating that the peroxisomal import of PTS2-containing proteins was to some extent impaired in *Tysnd1^−/−^* hepatocytes while accumulating in the cells. The overall accumulation of peroxisomal proteins may lead to the saturation of the Pex5- and Pex7-mediated peroxisomal protein transport capacity. RNAi knockdown of Tysnd1 in Hela cells reportedly [52] (data not shown) resulted in normal peroxisomal localization of Agps without apparent effect on the import system of peroxisomal proteins. We interpret the discrepancy between earlier reported results and ours, obtained from primary hepatocytes of *Tysnd1^−/−^* mice, as an effect of residual Tysnd1 protein expression after insufficient knock-down of Tysnd1 by RNAi.

In mammals the import of PTS2-containing proteins into the peroxisome depends on the binding to Pex7 and the direct interaction of Pex7-bound PTS2 protein with the long isoform of Pex5 (Pex5pL) [53], [54]. The N-terminal PTS2 signal is cleaved when the Pex5pL-Pex7-PTS2 protein complex has been transported into the peroxisome [9]. In *Pex7^−/−^* mice, Agps is absent in the liver and brain, suggesting that the precursor form of PTS2-containing proteins are unstable and prone to degradation [8] when they are not bound to Pex7. In contrast, the precursor form of Agps in *Tysnd1^−/−^* mice hepatocytes was not degraded (Figure 6G). These results strongly suggest that the binding of Pex7 to PTS2-containing proteins and subsequent association with Pex5pL in *Tysnd1^−/−^* mice is somehow impaired, which seems to affect Pex7 recycling and the degradation of PTS2-containing proteins [55]. Contrary to other models of peroxisome biogenesis disorders in which mislocalized peroxisomal proteins undergo accelerated degradation, the abundance of these proteins in our mouse model suggests that hitherto unidentified factors play a role in stabilizing these proteins and these will be the subject of future studies that may elucidate novel aspects about peroxisomal biogenesis.

The precise mechanism how the cleaved PTS2 signal is detached from Pex7 and Pex5pL is still unknown, but it might resemble the recently identified mechanism of Ubp15p-mediated Pex5 detachment in yeast from the PTS1 signal. Ubp15p, a ubiquitin hydrolase, cleaves off the ubiquitin moieties from the PTS1 receptor Pex5p [56]. The released Pex5p becomes available for a new round of matrix protein import from the cytosol [56].

**Figure 7:**
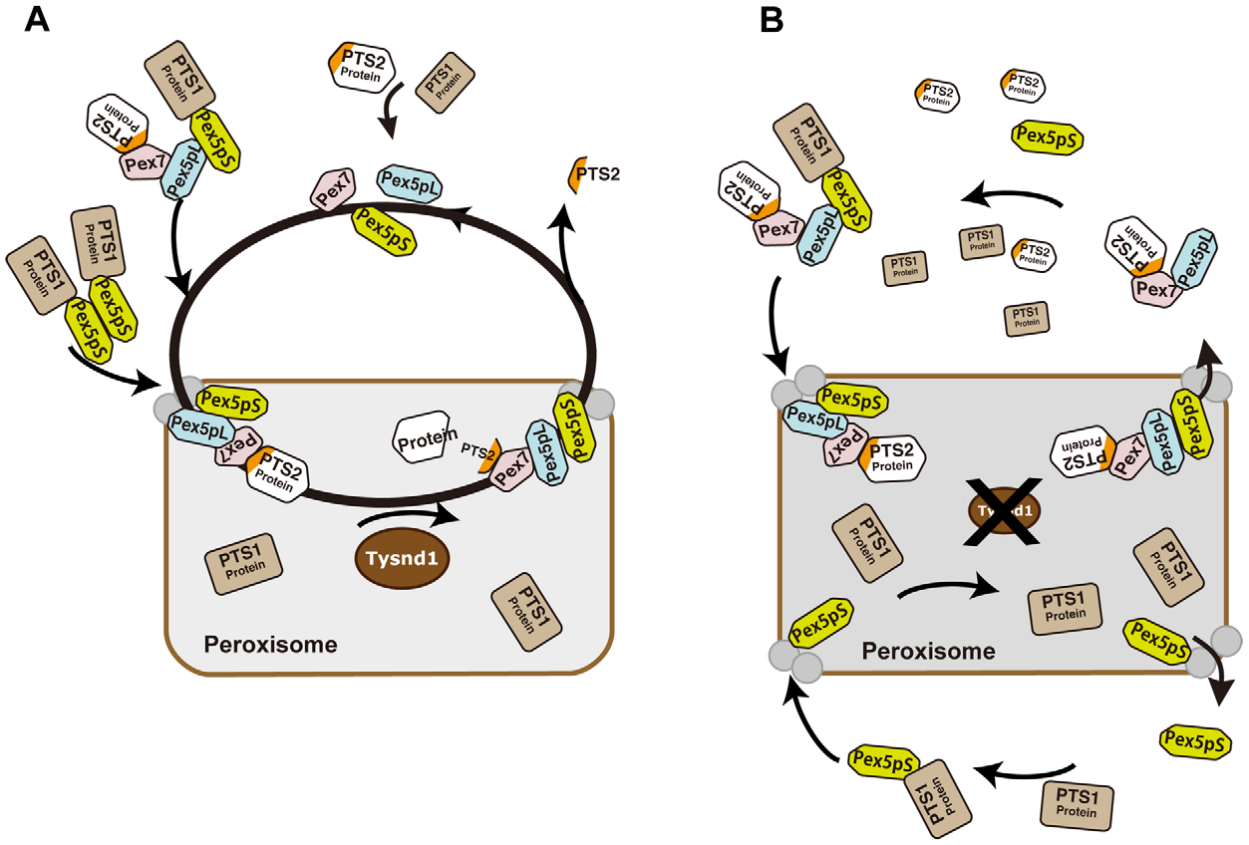
Proposed model of PTS2-protein import into peroxisomes in presence or absence of Tysnd1. A. Unprocessed PTS2 protein is imported from the cytoplasm into the peroxisome by Pex7 in association with the long isoform of Pex5 (Pex5pL). Tysnd1 processes the imported protein after the PTS2 signal and Pex7 and Pex5pL are recycled to the cytoplasm. The PTS2-containing fragment is exported from the peroxisome to the cytoplasm. Subsequent Pex5 and Pex7 docking components Pex13 and Pex14 are displayed as gray circles, but not individually labelled for reasons of simplicity. B. Tysnd1-deficient mouse peroxisome. Unprocessed PTS2-containing proteins remain bound as Pex7-Pex5pL cargo complex. Saturation of Pex7 and Pex5pL transport with relative shortage of free Pex5pL and Pex7 causes partial peroxisomal location appearance and accumulation of peroxisomal proteins outside the peroxisome.

Based on the current knowledge of Pex7- and Pex5pL- dependent PTS2-containing protein import into mammalian peroxisomes and the interference with the peroxisomal localization of Acaa1, Agps and Phyh caused by defective Tysnd1 processing, we propose a model which would leave, in the absence of Tysnd1-proteolytic removal of the PTS2 sequence, most PTS2-containing proteins bound to Pex7 in association with Pex5pL (Figure 7). The Pex7-Pex5pL-PTS2 protein complex returns to the cytosol, thereby limiting the rate of PTS2-containing protein import. Decreasing levels of free Pex5pL are predicted to affect also Pex5pL/Pex5pS heterodimerization and indirectly the import of PTS1-containing proteins by Pex5pL/Pex5pS heterodimeric oligomers [54], which may narrow PTS1 import to Pex5pS homodimers. As a result PTS1- and PTS2-containing peroxisomal matrix proteins would accumulate in the cytoplasm as seen in *Tysnd1^−/−^* mice.

In conclusion, *Tysnd1^−/−^* mice show reduced β-oxidation and phytanic acid metabolism. The changes in the plasmalogen composition, which we consider a contributing factor, but not necessarily the cause of male infertility in *Tynsd1^−/−^* mice are thought to be secondary effects of altered cellular acyl-CoA pools mediated by the reduced β-oxidation. Faulty peroxisomal targeting of novel Tysnd1 PTS2-containing substrates Phyh and Agps, and the previously reported substrate Acaa1 decreases their activities. Since Tysnd1 acts as a protease that affects the function of its substrates in the mouse model, we anticipate a new human peroxisomal disease entity caused by impaired TSYND1 functions that trigger a combination of mild dysfunctions among TYSND1 processing-dependent peroxisomal enzymes.

## Methods

### Generation of *Tysnd1* KO mice

Heterozygous *Tysnd1* knock-out mice were generated by TaconicArtemis (Cologne, Germany) under an ArteMice CONSTITUTIVE service contract. Briefly, genomic fragments of C57BL/6 BAC DNA (RP23-302P6) were subcloned into pTysnd1 FINAL Seq (MK141) vector. The deletion of exons 2 and 3 was confirmed by Southern blotting. C57BL/6N embryonic stem (ES) cells were electroporated with the construct and cultured. After confirming homologous recombination by Southern blotting, the ES cells were microinjected into C57BL/6J blastocysts. Germline transmission was achieved by crossing chimeric males with C57BL/6J females. Heterozygous mice breeding produced viable homozygous Tysnd1 null mice. Since the mice were of a hybrid C57BL/B6J, B6N background, heterozygous F1 mice were back-crossed for two generations with C57BL/6J mice. The mice were housed under specific pathogen free conditions at 23°C with a 12 h light-dark cycle. All mice used in this study were maintained and handled according to the protocols approved by the Animal Research Committee of Saitama Medical University.

Genotyping of male and female mice was performed by multiplex PCR under the following conditions: 5 min at 94°C; 35 cycles of 30 s at 94°C, 30 s at 55°C, 1 min at 72°C; and a final extension for 10 min at 72°C. The P1 forward primer (5′-cctggctcctcactgtttgc-3′) was combined with two reverse primers P2 (5′-ctacacttaaccagatgtgctttcc-3′) and P3 (5′-gtcatagtagtggccagaacc-3′). Primers P1 and P2 amplify the wild-type allele (237 bp amplicon). Primer pair P1 and P3 amplify the null allele with an amplicon size of 339 bp.

### RNA extraction and real-time RT–PCR

Livers of *Tysnd1^−/−^*, wild-type and heterozygote mice were immersed in RNA-later solution (Takara). Total RNA was isolated using the RNeasy Miniprep kit (Qiagen). Real-time RT-PCR was carried out using the same primers (Table S4) and method as described [14]

### Diet

Mice were fed *ad libitum* with standard rodent diet CE-2 (Clea Japan). During experiments with high-fat diet mice were given D12492 Rodent Diet containing 60 kcal% fat (Research Diets). Control mice were fed with D06041501 Rodent Diet containing 10 kcal% fat (Research Diets). The diet was changed one to two weeks prior to oral phytol administration from CE-2 to D06041501 control diet. Orally administered phytol (15 mg/day/mouse) was suspended in 0.25% sodium carboxy methyl cellulose.

### Japan Mouse Clinic (JMC) pipeline 1 and home cage activity

Fundamental and in depth screens of JMC pipeline 1 were performed with 7–26 weeks old mice as previously reported [17]. Modified-SHIRPA which is a component of JMC pipeline 1 was routinely conducted for eight weeks old mice. It involved 42 tests identical to the first stage of the original SHIRPA protocol [18]. Home cage locomotor activity, which is not included in JMC pipeline 1 was tested as part of an energy metabolism screen as previously described by Cao *et al.* [19].

### Antibodies

Custom-made rabbit polyclonal antibodies against Tysnd1 [14], Acaa1 also called prethiolase [14], Acox1, ScpX, Scp2, Hsd17b4 and Agps were obtained from Scrum. Anti-Phyh (2858-1-AP; Proteintech Group, Inc.), anti-Pex7 (20614-1-AP; Proteintech Group) and anti-Pex5 (GTX109798; GeneTex) polyclonal antibody were purchased from Funakoshi. Monoclonal anti-Pmp70 (SAB4200181; Sigma Aldrich) and rabbit anti-Gapdh antibodies (G9545; Sigma-Aldrich) were purchased from Sigma-Aldrich. The antigenic sequences and their positions are shown in Table S3. MN9 is a monoclonal antibody raised against mouse spermatozoa that has been previously characterized [57].

### Cloning of Tysnd1 substrate candidate genes

Protein-coding regions of potential Tysnd1 substrate candidate genes were amplified by PCR using cDNA products from a male liver of C57BL/6J mice, RIKEN Mouse Genome Encyclopedia DNAbook (DNAform). Primer information is shown in Table S4. For co-expression studies the PCR products of candidates were cloned into pcDNA3.1 or pcDNA3.1/V5-His-TOPO vectors (Invitrogen). For subcellular localization experiments the PCR products of candidates targeted by C-terminal PTS1 or N-terminal PTS2 were cloned into pcDNA3.1/NT-GFP-TOPO and pcDNA3.1/CT-GFP-TOPO vectors (Invitrogen), respectively. N-Tysnd1-Flag-C vector was used as previously described [14]

### Cell culture

COS7 cells were grown under 5% CO_2_ at 37°C in Dulbecco's modified Eagle's Medium (DMEM) (GIBCO) containing 10% (v/v) heat-inactivated fetal bovine serum (Bio West) and 0.1 mM non-essential amino acid (GIBCO). Primary cell cultures were prepared from livers of six weeks old male mice. Excised livers were drained of blood by flushing the hepatoportal vein with pre-circulation medium (HBSS(−) without phenol red, with HEPES, pH 7.2) and refluxed with circulation medium (1 mg/ml collagenase type IV in HBSS(−)). Once the liver texture became soft, tissue cells were suspended and incubated with circulation medium for 20 min at 37°C. The cell suspension was filtered through a 70 µm nylon mesh washed with DMEM and cultured with DMEM containing 10% FBS, 100 U/ml penicillin and 0.1 mg/ml streptomycin.

### Transfection

One day before transfection experiments, COS7 cells or primary cell cultures were seeded in plates and grown until 90–95% confluence. Transfections of COS7 cells and primary culture cells were performed according to the manufacturers' protocols with Lipofectamine 2000 (Invitrogen) and LTX (Invitrogen), respectively.

### Total protein extraction

Liver or testis samples were homogenized in radioimmunoprecipitation assay (RIPA) buffer containing protease inhibitor cocktail (Roche), followed by ultrasonic fragmentation and centrifugation.

### Separation of peroxisomal fractions using isotonic mitochondrial buffer

Peroxisomal fractions of liver were obtained as described by Omi *et al.* [58]. The cytosolic and peroxisomal fractions were confirmed by Western blotting using anti-Gapdh and anti-Pmp70 antibodies, respectively (Table S3).

### Western blotting

The protein extracts were separated by SDS-polyacrylamide gel electrophoresis and transferred to Immobilon-P membranes (Millipore) at least in duplicates. The Western blots were probed with anti-rabbit antibodies (see Antibodies) conjugated to anti-Rabbit IgG HRP (W401B31549303, Promega) or anti-mouse IgG-HRP ECL (NA931, GE Healthcare Life Sciences). The recognized proteins were detected by using ECL plus or ECL advance Western blotting detection kits (GE Healthcare Life Sciences).

### Measurement of β-oxidation activity

Livers of 15 weeks old male mice were excised and homogenized in 0.25 M sucrose and 5 mM MOPS using a Potter-Elvehjem homogenizer. After centrifugation of the homogenate at 3,000×g for 1 min the supernatant was incubated with 37°C for 60 min with reaction buffer as described elsewhere [59], [60]. [1–^14^C]lignoceric and [1–^14^C]palmitic acids (Muromachi Yakuhin) were used to measure peroxisomal and mitochondrial β-oxidation activities, respectively. The reaction was stopped by adding 1/5 volume of 1N KOH. Fatty acids were heated at 60°C and acidified in 6% perchloric acid for 1 hour on ice. After centrifugation at 7,700×g for 10 min, the [1–^14^C]lignoceric or [1–^14^C]palmitic acids were removed with 1.5 ml chloroform-methanol (2∶1) and ^14^C-labeled water-soluble metabolites were analyzed in a liquid scintillation counter (Beckman, LS 6500).

### Phenotypic analysis of sperms

Spermatic fluid was extracted from the caudal region of the epididymis. The samples were incubated for 15 min at 37°C with 1∶10 diluted mitochondrial stain MitoFluor Red 589 (Molecular Probes). After washing with 1×PBS the samples were mounted on glass slides using DAPI nuclear stain-containing mounting solution (Vector Laboratories). Digital images were taken with Axiovert 200 M (Zeiss).

### Measurement of plasmalogen species

Total lipids were extracted with chloroform/methanol (1∶2 by vol.) from freeze-dried mouse epididymides and testes. Plasmalogens were analyzed by liquid chromatography/electrospray ionization tandem mass spectrometry (LC/ESI-MS/MS). Liquid chromatography separation was performed using an Accela UPLC system (Thermo Fisher) with a BEH C8 column (1.7 µm, 100 mm×2.1 mm i.d.; Waters) at 60°C and a flow rate of 450 µl/min. Mobile phase A consisted of water containing 5 mM ammonium formate. Mobile phase B consisted of acetonitrile. MS analysis was performed using a TSQ Quantum Access Max (Thermo Fisher) equipped with an HESI probe in positive ion mode. The number of molecular species measured by LC-MS/MS was thirty-nine for both PlsCho and PlsEtn when limited to those containing at least one percent of total plasmalogens. The collision energy was 32 eV for PlsCho and 20 eV for PlsEtn.

### Paraffin-embedded sections

Tissue samples were immersed overnight in 4% paraformaldehyde in phosphate buffered saline (PBS, pH 7.4), washed with PBS and dehydrated in ethanol solutions containing 70% to 100% ethanol. The dehydrated samples were cleared in xylene/ethanol and xylene, and embedded in paraffin (Sakura Fine Tech Japan). All samples were cut into 8–10 µm sections using a large sliding microtome (Yamato Kohki) and mounted on glass slides.

### Hematoxylin-eosin (HE) staining of paraffin sections

The sections were deparaffinized by passing them through xylene, graded ethanol series (100–70%) and PBS. After staining with hematoxylin and eosin the sections were dehydrated in ethanol and embedded in glycerol.

### Electron microscopic (EM) analysis

EM analysis of sperm and testis samples derived from two *Tysnd1^−/−^* and two *Tysnd1^+/+^* ten weeks old male mice were performed as described before [61]. The liver samples were fixated by immersion in 2.5% glutaraldehyde plus 4% paraformaldehyde in 0.1 M phosphate buffer (pH 7.2) at 4°C for 2 hr, and postfixated for 1 h in a solution of 1% osmium tetroxide in phosphate buffer. The samples were then dehydrated in graded ethanol and embedded in epoxy resin. Ultrathin sections were cut with an ultramicrotome (Leica) at a thickness of 60 nm, mounted on 200-mesh nickel grids and counterstained with 2% uranyl acetate and 1% lead citrate. The sections were examined under a JEM 1010 transmission electron microscope (JEOL) with accelerating voltage of 80 kV.

### Measurement of VLCFA, phytanic and pristanic acid levels

Blood serum VLCFAs and phytanic acid levels *Tysnd1^−/−^* and wild-type mice were measured by using GS/MS as previously reported [62]. VLCFAs were determined from the serum of ten weeks old mice. Phytanic acid was measured in the serum of both ten and 38–39 weeks old mice. Plasma pristanic acid levels of ten weeks old male *Tysnd1^−/−^* and wild-type mice were determined by UPLC-MS/MS as described in Text S1.

### Analysis of subcellular protein localization by confocal laser-scanning microscopy

Primary hepatocytes of six weeks old male *Tysnd1* ^+/+^ or *Tysnd1* ^−/−^ were cultured on cover glasses in 6-well plates and each of the GFP-fused Tysnd1 substrate expression vector (Table S4) was either singly transfected or co-transfected with the C-terminal PTS1-containing peroxisomal marker DsRed2-Peroxi (Clontech). All transfections except for Acaa1-GFP and GFP-ScpX were performed in duplicate. Twenty-four to 36 hours after transfection the living cells were analyzed. The cells used for immunostaining were first fixed in 4% paraformaldehyde containing PBS, then blocked in 2% skim milk and incubated with the primary antigen and anti-Pmp70 (peroxisomal membrane marker). Immune complexes were visualized using Alexa Fluor 568 or Alexa Fluor 488 labeled goat anti-rabbit IgG antibodies (Molecular Probes). Cells were observed with a TCS SP2 confocal laser-scanning microscope (Leica). The fluorescence of GFP or Alexa Fluor 488 was measured at 488 nm excitation. DsRed2-Peroxi and Alexa Fluor 568 fluorescence was measured at 543 nm excitation.

## Supporting Information

Figure S1 Anthropometrical parameters. Body weight (A), length (B) and body mass index (BMI) (C) of male mice. CD indicates mice fed with control D06041501 rodent diet containing 10 kcal% fat (Research Diets, Inc.). HFD indicates mice on high fat D12492 rodent diet containing 60 kcal% fat (Research Diets, Inc.). Each error bar represents the mean ± SE in n = 9–15.
(PDF)

Figure S2 Peroxisome proliferation occurred in *Tysnd1^−/−^* mice. A. EM image analysis of liver samples taken from male *Tysnd1* ^−/−^ and *Tysnd1* ^+/+^ mice fed with control diet. Arrows indicate the peroxisome. Scale bar: 2 µm. B and C. Number (count/field (1,000 µm^2^)) and size (% area of field) of peroxisomes analyzed in EM images. Error bars represents the mean ± SE of n = 3–5. **p<0.01.
(PDF)

Figure S3 Total plasmalogens in testes of male Tysnd1^−/−^ and Tysnd1^+/+^ mice. Total plasmalogens were analyzed by liquid chromatography/electrospray ionization tandem mass spectrometry (LC/ESI-MS/MS).
(PDF)

Figure S4 Phytol feeding experiment. A. Ratio of phytanic acid (C20∶0-branched) to C16∶0 (GC-MS/MS) in blood serum of 38–39 weeks old male mice. Error bars represents the mean ± SE of n = 5. B. Body weight change of male *Tysnd1* ^−/−^ mice and wild-type mice during 13 days of oral administration of phytol and 0.5% sodium carboxy methyl cellulose (CMC) without phytol. At day 0 of the phytol feeding experiment the mice were eight weeks old. Error bars represents the mean ± SE of n = 4–9. C. Body weight rate increase or decrease after 13 days of phytol diet. Error bars represents the mean ± SE of n = 4–9. ***p<0.001. D. Total liver fat in ten weeks old male *Tysnd1* ^−/−^ and wild-type mice after oral administration of phytol and 0.5% sodium carboxy methyl cellulose (CMC) without phytol. Each error bar represents the mean ± SE of n = 5. *p<0.05. E. Triglyceride assay of liver total fat for ten weeks old male *Tysnd1* ^−/−^ and wild-type mice after oral administration of phytol and without 0.5% sodium carboxy methyl cellulose (CMC) without phytol. NS indicates not significant. F. The mitochondrial β-oxidation activity as determined by [1–^14^C]palmitic acids did not differ between ten weeks old *Tysnd1* ^−/−^ and wild-type mice after oral administration of phytol and 0.5% sodium carboxy methyl cellulose (CMC) without phytol. Each error bar represents the mean ± SE of n = 3–6. NS indicates not significant. G. Plasma pristanic acid (C19∶0-branched) concentration (µg/ml) measured by UPLC-MS/MS in ten weeks old male mice. Error bars represents the mean ± SE of n = 3.
(PDF)

Figure S5 Effect of Tysnd1 expression on processing its candidate substrates. COS7 cells were transiently co-transfected with the indicated combinations of HA-Gnpat (A), Far1-V5 (B), Far2-V5 (C) and HA-Amacr (D) and Tysnd1 expression plasmids.
(PDF)

Figure S6 PTS2-containing proteins are imported into peroxisomes of *Tysnd1* ^−/−^ primary hepatocytes. The signal intensity of GFP and DsRed2 fluorescence corresponding to the confocal-scanned images (Figure 6) was measured along the green line. Tysnd1 substrates expressed as GFP fusion proteins are shown in green peroxisome-specific DsRed2-Peroxi in red. The localization of PTS2-containing proteins Acaa1(A), Phyh(B) and Agps(C) poorly overlaps with that of DsRed2-Peroxi, while the localization of PTS1-containing proteins Acox1(D), Hsd17b4(E) and ScpX(F) coincides with DsRed2-Peroxi.
(PDF)

Figure S7 Subcellular localization of peroxisomal proteins in primary hepatocytes of *Tysnd1^−/−^* and *Tysnd1^+/+^* mice. As a control (A) we transfected the cells with DsRed2-Peroxi and immunostained with anti-Pmp70 plus Alex Fluor 488 (green). Acaa1-GFP (B), Phyh-GFP (C), GFP-Hsd17b4 (D) and Acaa1-GFP co-transfected with HA-Acox1 (E) are shown in green and peroxisomal membrane marker Pmp70 in red after immunostaining with anti-Pmp70 and Alexa Fluor 568.
(PDF)

Table S1 A. Comparison of *Tysnd1^−/−^* and *Tysnd1^+/−^* pregnancies and littermate size. B. *In vitro* fertilization rates of *Tysnd1^−/−^* and *Tysnd1^+/−^* oocytes.
(PDF)

Table S2 Clinical blood serum biochemical analyses of *Tysnd1^−/−^* and *Tysnd1^+/+^* mice administered with phytol.
(PDF)

Table S3 Polyclonal and monoclonal antibodies and their antigenic peptides.
(PDF)

Table S4 Primer sequences used for real time RT-PCR and constructing pcDNA3.1-V5, -HA and -GFP expression vectors.
(PDF)

Text S1 Supporting Methods.
(PDF)

## References

[1] Wanders RJ, Vreken P, Ferdinandusse S, Jansen GA, Waterham HR, et al (2001) Peroxisomal fatty acid alpha- and beta-oxidation in humans: enzymology, peroxisomal metabolite transporters and peroxisomal diseases. Biochem Soc Trans 29: 250–267. 11356164 10.1042/0300-5127:0290250

[2] Sztriha L, Al-Gazali LI, Wanders RJ, Ofman R, Nork M, et al (2000) Abnormal myelin formation in rhizomelic chondrodysplasia punctata type 2 (DHAPAT-deficiency). Dev Med Child Neurol 42: 492–495. 10972423 10.1017/s0012162200000918

[3] Steinberg SJ, Dodt G, Raymond GV, Braverman NE, Moser AB, et al (2006) Peroxisome biogenesis disorders. Biochim Biophys Acta 1763: 1733–1748. 17055079 10.1016/j.bbamcr.2006.09.010

[4] Santos MJOJ, Garrido J, Leighton F (1985) Peroxisomal organization in normal and cerebrohepatorenal (Zellweger) syndrome fibroblasts. Proc Natl Acad Sci U S A 82: 6556–6560. 2995971 10.1073/pnas.82.19.6556 PMC391248

[5] Arias JA, Moser AB, Goldfischer SL (1985) Ultrastructural and cytochemical demonstration of peroxisomes in cultured fibroblasts from patients with peroxisomal deficiency disorders. J Cell Biol 100: 1789–1792. 3988808 10.1083/jcb.100.5.1789 PMC2113858

[6] Suzuki Y, Shimozawa N, Orii T, Igarashi N, Kono N, Hashimoto T (1988) Molecular analysis of peroxisomal beta-oxidation enzymes in infants with Zellweger syndrome and Zellweger-like syndrome: further heterogeneity of the peroxisomal disorder. Clin Chim Acta 172: 65–76. 2452040 10.1016/0009-8981(88)90121-0

[7] Tager JM, Van der Beek WA, Wanders RJ, Hashimoto T, Heymans HS, et al (1985) Peroxisomal beta-oxidation enzyme proteins in the Zellweger syndrome. Biochem Biophys Res Commun 126: 1269–1275. 3977916 10.1016/0006-291x(85)90322-5

[8] Brites P, Motley AM, Gressens P, Mooyer PA, Ploegaert I, et al (2003) Impaired neuronal migration and endochondral ossification in Pex7 knockout mice: a model for rhizomelic chondrodysplasia punctata. Hum Mol Genet 12: 2255–2267. 12915479 10.1093/hmg/ddg236

[9] Mukai S, Fujiki Y (2006) Molecular mechanisms of import of peroxisome-targeting signal type 2 (PTS2) proteins by PTS2 receptor Pex7p and PTS1 receptor Pex5pL. J Biol Chem 281: 37311–37320. 17040904 10.1074/jbc.M607178200

[10] Subramani S, Koller A, Snyder WB (2000) Import of peroxisomal matrix and membrane proteins. Annu Rev Biochem 69: 399–418. 10966464 10.1146/annurev.biochem.69.1.399

[11] Gould SJ, Keller GA, Hosken N, Wilkinson J, Subramani S (1989) A conserved tripeptide sorts proteins to peroxisomes. J Cell Biol 108: 1657–1664. 2654139 10.1083/jcb.108.5.1657 PMC2115556

[12] Neuberger G, Maurer-Stroh S, Eisenhaber B, Hartig A, Eisenhaber F (2003) Motif refinement of the peroxisomal targeting signal 1 and evaluation of taxon-specific differences,. J Mol Biol 328: 567–579. 12706717 10.1016/s0022-2836(03)00318-8

[13] Petriv OI, Tang L, Titorenko VI, Rachubinski RA (2004) A new definition for the consensus sequence of the peroxisome targeting signal type 2. J Mol Biol 341: 119–134. 15312767 10.1016/j.jmb.2004.05.064

[14] Kurochkin IV, Mizuno Y, Konagaya A, Sakaki Y, Schönbach C, et al (2007) Novel peroxisomal protease Tysnd1 processes PTS1- and PTS2-containing enzymes involved in beta-oxidation of fatty acids. EMBO J 26: 835–845. 17255948 10.1038/sj.emboj.7601525 PMC1794383

[15] Ohba T, Holt JA, Billheimer JT, Strauss JF 3rd (1995) Human sterol carrier protein x/sterol carrier protein 2 gene has two promoters. Biochemistry 34: 10660–10668. 7654720 10.1021/bi00033a042

[16] Ossendorp BC, Van Heusden GP, De Beer AL, Bos K, Schouten GL, et al (1991) Identification of the cDNA clone which encodes the 58-kDa protein containing the amino acid sequence of rat liver non-specific lipid-transfer protein (sterol-carrier protein 2). Homology with rat peroxisomal and mitochondrial 3-oxoacyl-CoA thiolases. Eur J Biochem 201: 233–239. 1915369 10.1111/j.1432-1033.1991.tb16279.x

[17] Wakana S, Suzuki T, Furuse T, Kobayashi K, Miura I, et al (2009) Introduction to the Japan Mouse Clinic at the RIKEN BioResource Center. Exp Anim 58: 443–450. 19897927 10.1538/expanim.58.443

[18] Masuya H, Inoue M, Wada Y, Shimizu A, Nagano J, et al (2005) Implementation of the modified-SHIRPA protocol for screening of dominant phenotypes in a large-scale ENU mutagenesis program. Mamm Genome 11: 829–837. 16284798 10.1007/s00335-005-2430-8

[19] Cao Y, Nakata M, Okamoto S, Takano E, Yada T, et al (2011) PDK1-Foxo1 in agouti-related peptide neurons regulates energy homeostasis by modulating food intake and energy expenditure. PLoS ONE 6: e18324 doi:10.1371/journal.pone.0018324. 21694754 10.1371/journal.pone.0018324 PMC3072380

[20] Martínez P, Morros A (1996) Membrane lipid dynamics during human sperm capacitation. Front Biosci 1: d103–117. 9159218 10.2741/a119

[21] Nagan N, Zoeller RA (2001) Plasmalogens: biosynthesis and functions. Prog Lipid Res 40: 199–229. 11275267 10.1016/s0163-7827(01)00003-0

[22] Huyghe S, Mannaerts GP, Baes M, Van Veldhoven PP (2006) Peroxisomal multifunctional protein-2: The enzyme, the patients and the knockout mouse model. Biochim Biophys Acta 1761: 973–994. 16766224 10.1016/j.bbalip.2006.04.006

[23] Atshaves BPMA, Landrock D, Payne HR, Mackie JT, et al (2007) Effect of SCP-x gene ablation on branched-chain fatty acid metabolism. Am J Physiol Gastrointest Liver Physiol 292: G939–951. 17068117 10.1152/ajpgi.00308.2006

[24] Huyghe S, Schmalbruch H, Hulshagen L, Veldhoven PV, Baes M, et al (2006) Peroxisomal multifunctional protein-2 deficiency causes motor deficits and glial lesions in the adult central nervous system. Am J Pathol 168: 1321–1334. 16565505 10.2353/ajpath.2006.041220 PMC1606565

[25] Ferdinandusse S, Denis S, Clayton PT, Graham A, Rees JE, et al (2000) Mutations in the gene encoding peroxisomal alpha-methylacyl-CoA racemase cause adult-onset sensory motor neuropathy. Nat Genet 24: 188–191. 10655068 10.1038/72861

[26] Cheng JB, Russell DW (2004) Mammalian wax biosynthesis. I. Identification of two fatty acyl-Coenzyme A reductases with different substrate specificities and tissue distributions. J Biol Chem 37789–37797. 15220348 10.1074/jbc.M406225200 PMC2757098

[27] Kashiwayama Y, Asahina K, Shibata H, Morita M, Muntau AC, et al (2005) Role of Pex19p in the targeting of PMP70 to peroxisome. Biochim Biophys Acta 1746: 116–128. 16344115 10.1016/j.bbamcr.2005.10.006

[28] Fang Y, Morrell JC, Jones JM, Gould SJ (2004) PEX3 functions as a PEX19 docking factor in the import of class I peroxisomal membrane proteins. J Cell Biol 164: 863–875. 15007061 10.1083/jcb.200311131 PMC2172291

[29] Braverman N, Steel G, Obie C, Moser A, Moser H, et al (1997) Human PEX7 encodes the peroxisomal PTS2 receptor and is responsible for rhizomelic chondrodysplasia punctata. Nat Genet 15: 369–376. 9090381 10.1038/ng0497-369

[30] Motley AM, Hettema EH, Hogenhout EM, Brites P, ten Asbroek AL, et al (1997) Rhizomelic chondrodysplasia punctata is a peroxisomal protein targeting disease caused by a non-functional PTS2 receptor. Nat Genet 15: 377–380. 9090382 10.1038/ng0497-377

[31] Purdue PE, Zhang JW, Skoneczny M, Lazarow PB (1997) Rhizomelic chondrodysplasia punctata is caused by deficiency of human PEX7, a homologue of the yeast PTS2 receptor. Nat Genet 15: 381–384. 9090383 10.1038/ng0497-381

[32] Rodemer C, Thai TP, Brugger B, Kaercher T, Werner H, et al (2003) Inactivation of ether lipid biosynthesis causes male infertility, defects in eye development and optic nerve hypoplasia in mice. Hum Mol Genet 12: 1881–1895. 12874108 10.1093/hmg/ddg191

[33] ten Brink HJ, Stellaard F, van den Heuvel CM, Kok RM, Schor DS, et al (1992) Pristanic acid and phytanic acid in plasma from patients with peroxisomal disorders: stable isotope dilution analysis with electron capture negative ion mass fragmentography. J Lipid Res 33: 41–47. 1372637

[34] Bams-Mengerink AM, Majoie CB, Duran M, Wanders RJ, Van Hove J, et al (2006) MRI of the brain and cervical spinal cord in rhizomelic chondrodysplasia punctata. Neurology 66: 798–803. 16567694 10.1212/01.wnl.0000205594.34647.d0

[35] Alberts B, Johnson A, Lewis J, Raff M, Roberts K, et al.. (2002) Molecular Biology of the Cell, 4th edition, Chapter 12, Peroxisomes. . New York: Garland Science.

[36] Nolan JP, Hammerstedt RH (1997) Regulation of membrane stability and the acrosome reaction in mammalian sperm. FASEB J 11: 670–682. 9240968 10.1096/fasebj.11.8.9240968

[37] Brites P, Waterham HR, Wanders RJ (2004) Functions and biosynthesis of plasmalogens in health and disease. Biochim Biophys Acta 1636: 219–231. 15164770 10.1016/j.bbalip.2003.12.010

[38] Gorgas K, Teigler A, Komljenovic D, Just WW (2006) The ether lipid-deficient mouse: tracking down plasmalogen functions. Biochim Biophys Acta 1763: 1511–1526. 17027098 10.1016/j.bbamcr.2006.08.038

[39] Aveldaño MI, Rotstein NP, Vermouth NT (1992) Lipid remodelling during epididymal maturation of rat spermatozoa. Enrichment in plasmenylcholines containing long-chain polyenoic fatty acids of the n-9 series. Biochem J 283: 235–241. 1567371 10.1042/bj2830235 PMC1131019

[40] de Vet EC, Ijlst L, Oostheim W, Wanders RJ, van den Bosch H (1998) Alkyl-dihydroxyacetonephosphate synthase. Fate in peroxisome biogenesis disorders and identification of the point mutation underlying a single enzyme deficiency. J Biol Chem 273: 10296–10301. 9553082 10.1074/jbc.273.17.10296

[41] Goodenowe DB, Cook LL, Liu J, Lu Y, Jayasinghe DA, et al (2007) Peripheral ethanolamine plasmalogen deficiency: a logical causative factor in Alzheimer's disease and dementia. J Lipid Res 48: 2485–2498. 17664527 10.1194/jlr.P700023-JLR200

[42] Grimm MO, Kuchenbecker J, Rothhaar TL, Grösgen S, Hundsdörfer B, et al (2011) Plasmalogen synthesis is regulated via alkyl-dihydroxyacetonephosphate-synthase by amyloid precursor protein processing and is affected in Alzheimer's disease. J Neurochem 116: 916–925. 21214572 10.1111/j.1471-4159.2010.07070.x

[43] Braverman N, Chen L, Lin P, Obie C, Steel G, et al (2002) Mutation analysis of PEX7 in 60 probands with rhizomelic chondrodysplasia punctata and functional correlations of genotype with phenotype. Hum Mutat 20: 284–297. 12325024 10.1002/humu.10124

[44] Zhang X, Tanaka N, Nakajima T, Kamijo Y, Gonzalez FJ, et al (2006) Peroxisome proliferator-activated receptor a-independent peroxisome proliferation. Biochem Biophys Res Commun 350: 370–376. 16806075 10.1016/j.bbrc.2006.06.042

[45] Ferdinandusse S, Zomer AW, Komen JC, van den Brink CE, Thanos M, et al (2008) Ataxia with loss of Purkinje cells in a mouse model for Refsum disease. Proc Natl Acad Sci U S A 105: 17712–17717. 19004801 10.1073/pnas.0806066105 PMC2584743

[46] Mukherji M, Chien W, Kershaw NJ, Clifton IJ, Schofield CJ, et al (2001) Structure-function analysis of phytanoyl-CoA 2-hydroxylase mutations causing Refsum's disease. Hum Mol Genet 10: 1971–1982. 11555634 10.1093/hmg/10.18.1971

[47] Manjithaya R, Nazarko TY, Farré JC, Subramani S (2010) Molecular mechanism and physiological role of pexophagy. FEBS Lett 584: 1367–1373. 20083110 10.1016/j.febslet.2010.01.019 PMC2843806

[48] Nazarko TY, Farré JC, Subramani S (2009) Peroxisome size provides insights into the function of autophagy-related proteins. Mol Biol Cell 20: 3828–3839. 19605559 10.1091/mbc.E09-03-0221 PMC2735482

[49] Mönnig G, Wiekowski J, Kirchhof P, Stypmann J, Plenz G, et al (2004) Phytanic acid accumulation is associated with conduction delay and sudden cardiac death in sterol carrier protein-2/sterol carrier protein-x deficient mice. J Cardiovasc Electrophysiol 15: 1310–1316. 15574183 10.1046/j.1540-8167.2004.03679.x

[50] Mackie JT, Atshaves BP, Payne HR, McIntosh AL, Schroeder F, et al (2009) Phytol-induced hepatotoxicity in mice. Toxicol Pathol 37: 201–208. 19188468 10.1177/0192623308330789 PMC2838495

[51] Biermann J, van den Bosch H (1999) In Vitro Processing of the Human Alkyldihydroxyacetonephosphate Synthase Precursor. Arch Biochem Biophys 368: 139–146. 10415121 10.1006/abbi.1999.1281

[52] Okumoto K, Kametani Y, Fujiki Y (2011) Two proteases, trypsin domain-containing 1 (Tysnd1) and peroxisomal lon protease (PsLon), cooperatively regulate fatty acid β-oxidation in peroxisomal matrix. J Biol Chem 286: 44367–44379. 22002062 10.1074/jbc.M111.285197 PMC3247999

[53] Braverman N, Dodt G, Gould SJ, Valle D (1998) An isoform of pex5p, the human PTS1 receptor, is required for the import of PTS2 proteins into peroxisomes. Hum Mol Genet 7: 1195–1205. 9668159 10.1093/hmg/7.8.1195

[54] Otera H, Harano T, Honsho M, Ghaedi K, Mukai S, et al (2000) The mammalian peroxin Pex5pL, the longer isoform of the mobile peroxisome targeting signal (PTS) type 1 transporter, translocates the Pex7p.PTS2 protein complex into peroxisomes via its initial docking site, Pex14p. J Biol Chem 275: 21703–21714. 10767286 10.1074/jbc.M000720200

[55] Nair DM, Purdue PE, Lazarow PB (2004) Pex7p translocates in and out of peroxisomes in Saccharomyces cerevisiae. J Cell Biol 167: 599–604. 15545321 10.1083/jcb.200407119 PMC2172567

[56] Debelyy MO, Platta HW, Saffian D, Hensel A, Thoms S, et al (2011) Ubp15p, a ubiquitin hydrolase associated with the peroxisomal export machinery. J Biol Chem 286: 28223–28234. 21665945 10.1074/jbc.M111.238600 PMC3151067

[57] Toshimori K, Tanii I, Araki S, Oura C (1992) Characterization of the antigen recognized by a monoclonal antibody MN9: unique transport pathway to the equatorial segment of sperm head during spermiogenesis. Cell Tissue Res 270: 459–468. 1486600 10.1007/BF00645047

[58] Omi S, Nakata R, Okamura-Ikeda K, Konishi H, Taniguchi H (2008) Contribution of peroxisome-specific isoform of Lon protease in sorting PTS1 proteins to peroxisomes. J Biochem 143: 649–660. 18281296 10.1093/jb/mvn020

[59] Erol E, Kumar LS, Cline GW, Shulman GI, Kelly DP, et al (2004) Liver fatty acid binding protein is required for high rates of hepatic fatty acid oxidation but not for the action of PPARalpha in fasting mice. FASEB J 18: 347–349. 14656998 10.1096/fj.03-0330fje

[60] Watkins PA, Ferrell EV Jr, Pedersen JI, Hoefler G (1991) Peroxisomal fatty acid beta-oxidation in HepG2 cells. Arch Biochem Biophys 289: 329–336. 1654856 10.1016/0003-9861(91)90419-j

[61] Nakamura T, Yao R, Ogawa T, Suzuki T, Ito C, et al (2004) Oligo-astheno-teratozoospermia in mice lacking Cnot7, a regulator of retinoid X receptor beta. Nat Genet 36: 528–533. 15107851 10.1038/ng1344

[62] Takemoto Y, Suzuki Y, Horibe R, Shimozawa N, Wanders RJ, et al (2003) Gas chromatography/mass spectrometry analysis of very long chain fatty acids, docosahexaenoic acid, phytanic acid and plasmalogen for the screening of peroxisomal disorders. Brain and Development 25: 481–487. 13129591 10.1016/s0387-7604(03)00033-0

