# Supplementary figures and images for: Tysnd1 Deficiency in Mice Interferes with the Peroxisomal Localization of PTS2 Enzymes, Causing Lipid Metabolic Abnormalities and Male Infertility
Source: PLoS Genet. 2013 Feb 14;9(2):e1003286. doi: 10.1371/journal.pgen.1003286 (PMC3573110; doi:10.1371/journal.pgen.1003286)

Figure S1

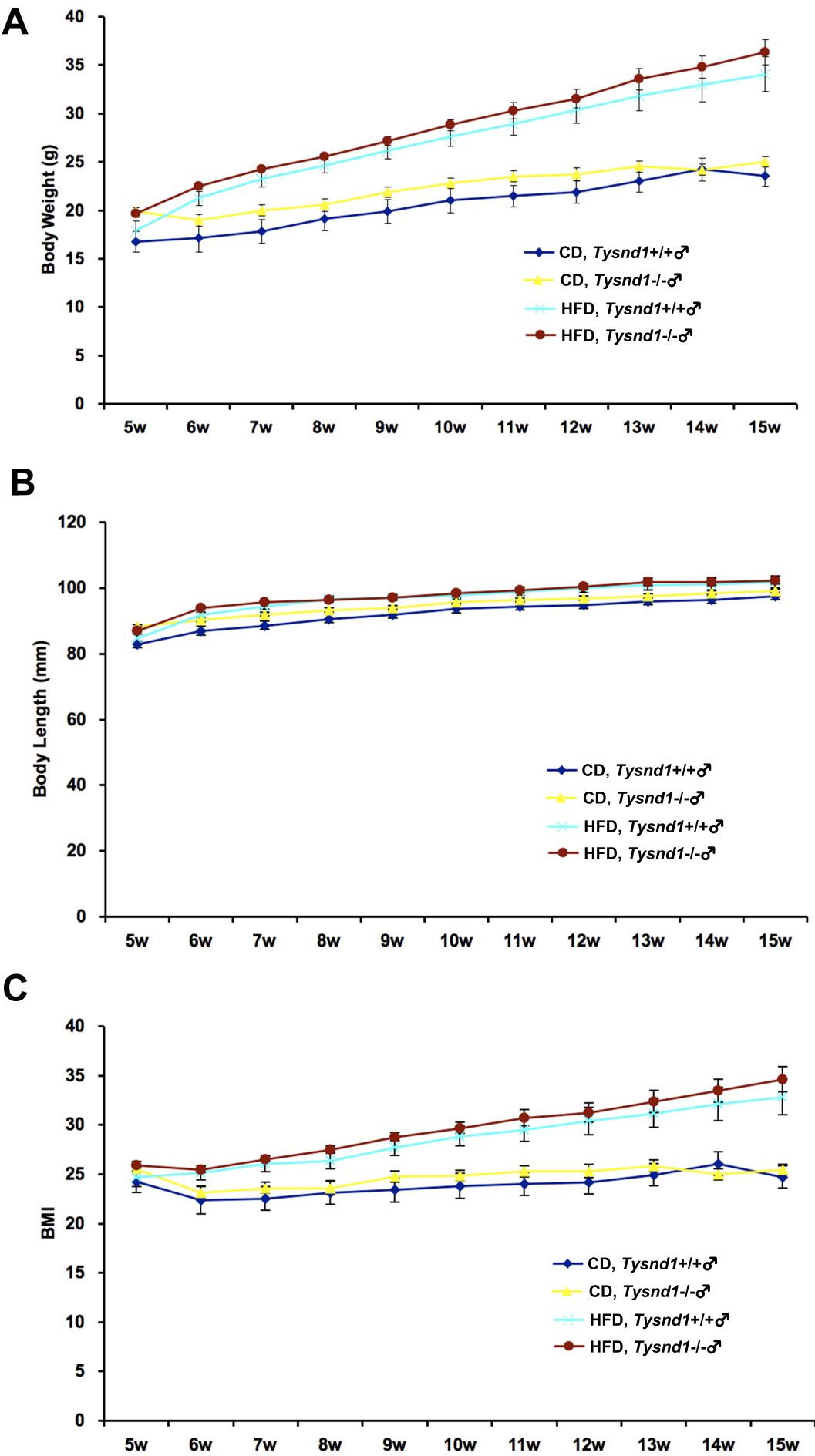

Supplement: Figure S1 — Anthropometrical parameters. Body weight (A), length (B) and body mass index (BMI) (C) of male mice. CD indicates mice fed with control D06041501 rodent diet containing 10 kcal% fat (Research Diets, Inc.). HFD indicates mice on high fat D12492 rodent diet containing 60 kcal% fat (Research Diets, Inc.). Each error bar represents the mean ± SE in n = 9–15. (PDF) [file pgen.1003286.s001.pdf]

**Figure S2**

**A**

*Tysnd1*<sup>+/+</sup> ♂

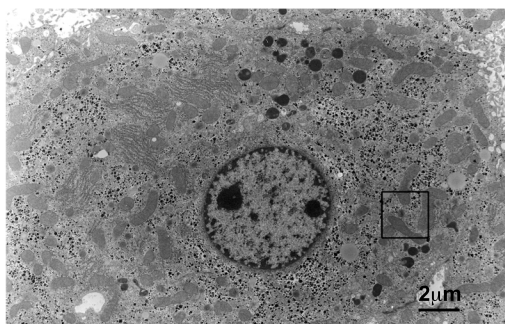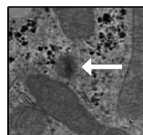

*Tysnd1*<sup>-/-</sup> ♂

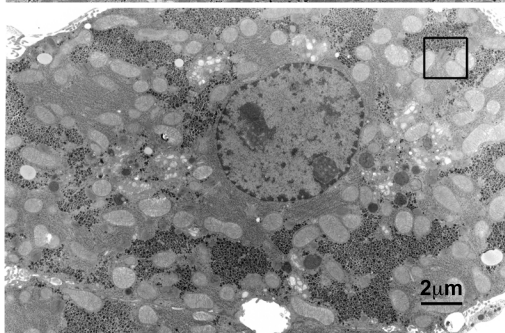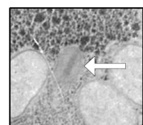

**B**

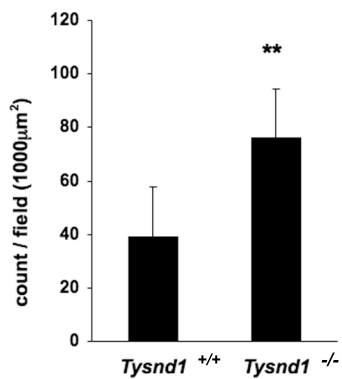

**C**

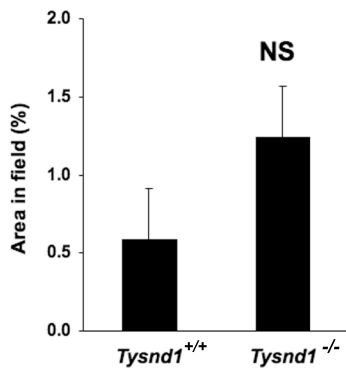

Supplement: Figure S2 — Peroxisome proliferation occurred in Tysnd1−/− mice. A. EM image analysis of liver samples taken from male Tysnd1 −/− and Tysnd1 +/+ mice fed with control diet. Arrows indicate the peroxisome. Scale bar: 2 µm. B and C. Number (count/field (1,000 µm2)) and size (% area of field) of peroxisomes analyzed in EM images. Error bars represents the mean ± SE of n = 3–5. **p<0.01. (PDF) [file pgen.1003286.s002.pdf]

**Figure S3**

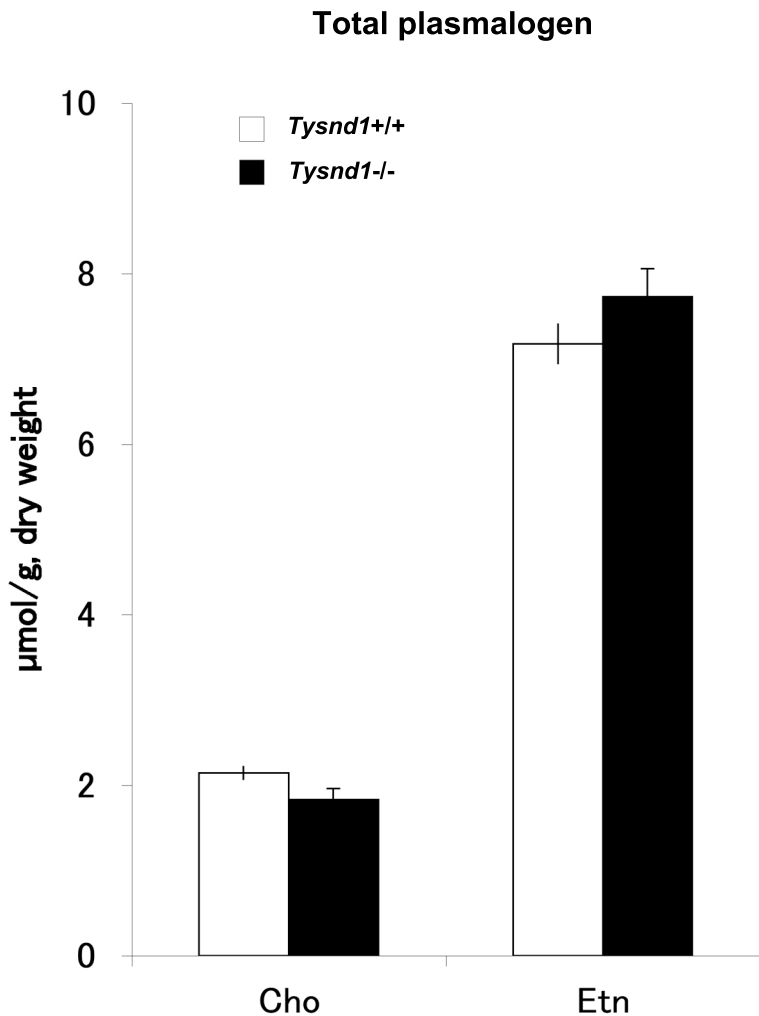

Supplement: Figure S3 — Total plasmalogens in testes of male Tysnd1−/− and Tysnd1+/+ mice. Total plasmalogens were analyzed by liquid chromatography/electrospray ionization tandem mass spectrometry (LC/ESI-MS/MS). (PDF) [file pgen.1003286.s003.pdf]

**Figure S4**

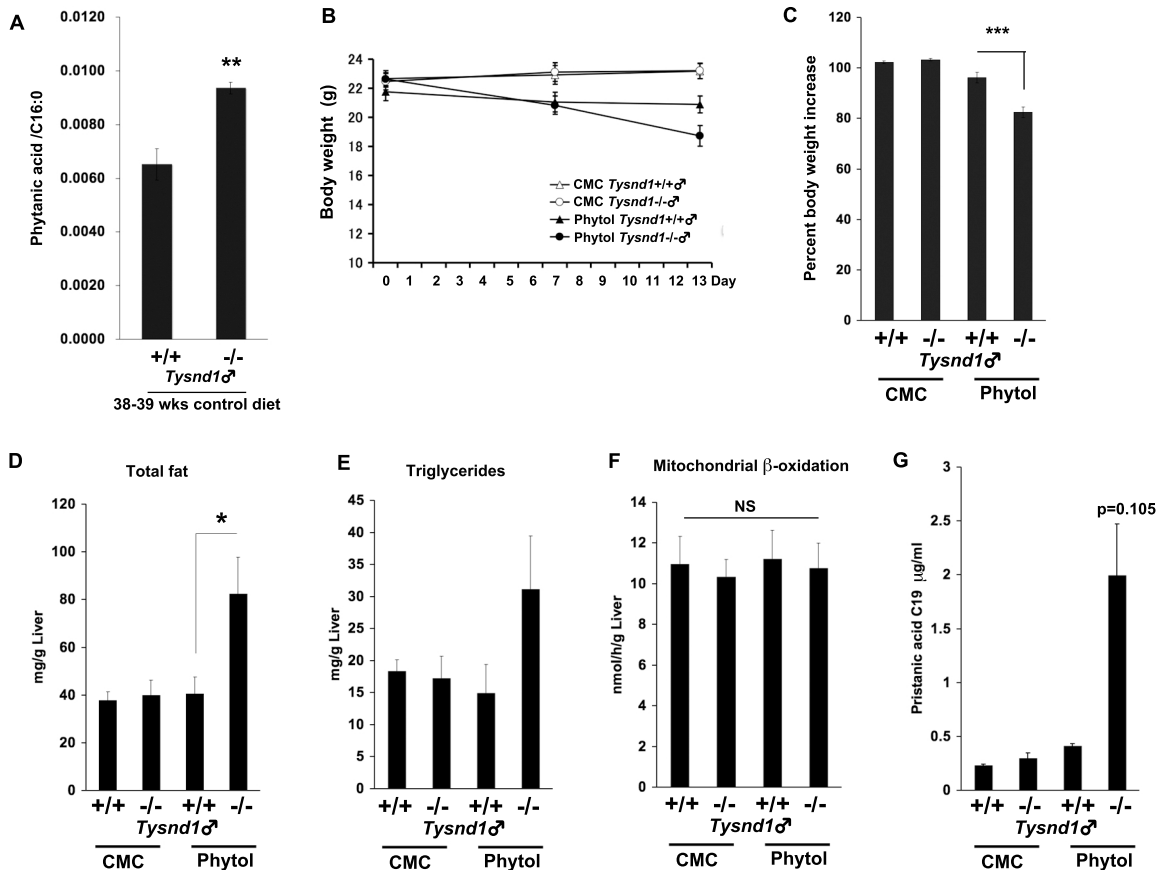

Supplement: Figure S4 — Phytol feeding experiment. A. Ratio of phytanic acid (C20∶0-branched) to C16∶0 (GC-MS/MS) in blood serum of 38–39 weeks old male mice. Error bars represents the mean ± SE of n = 5. B. Body weight change of male Tysnd1 −/− mice and wild-type mice during 13 days of oral administration of phytol and 0.5% sodium carboxy methyl cellulose (CMC) without phytol. At day 0 of the phytol feeding experiment the mice were eight weeks old. Error bars represents the mean ± SE of n = 4–9. C. Body weight rate increase or decrease after 13 days of phytol diet. Error bars represents the mean ± SE of n = 4–9. ***p<0.001. D. Total liver fat in ten weeks old male Tysnd1 −/− and wild-type mice after oral administration of phytol and 0.5% sodium carboxy methyl cellulose (CMC) without phytol. Each error bar represents the mean ± SE of n = 5. *p<0.05. E. Triglyceride assay of liver total fat for ten weeks old male Tysnd1 −/− and wild-type mice after oral administration of phytol and without 0.5% sodium carboxy methyl cellulose (CMC) without phytol. NS indicates not significant. F. The mitochondrial β-oxidation activity as determined by [1–14C]palmitic acids did not differ between ten weeks old Tysnd1 −/− and wild-type mice after oral administration of phytol and 0.5% sodium carboxy methyl cellulose (CMC) without phytol. Each error bar represents the mean ± SE of n = 3–6. NS indicates not significant. G. Plasma pristanic acid (C19∶0-branched) concentration (µg/ml) measured by UPLC-MS/MS in ten weeks old male mice. Error bars represents the mean ± SE of n = 3. (PDF) [file pgen.1003286.s004.pdf]

**Figure S5**

**A**

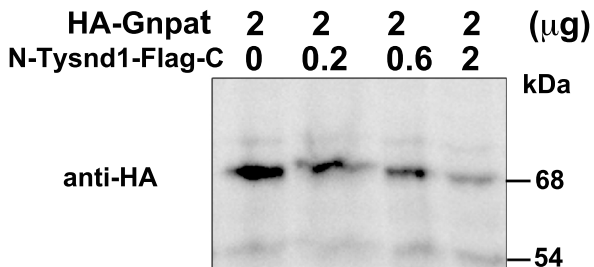

**C**

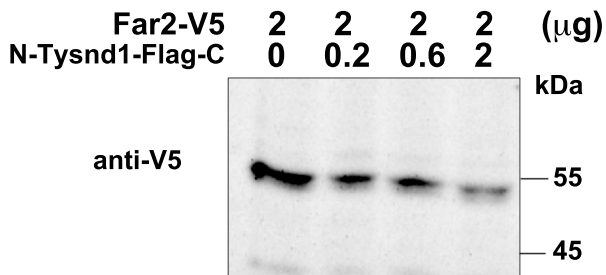

**B**

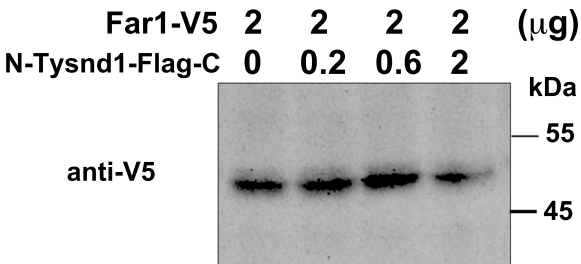

**D**

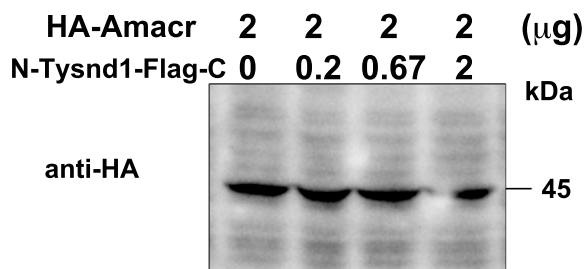

Supplement: Figure S5 — Effect of Tysnd1 expression on processing its candidate substrates. COS7 cells were transiently co-transfected with the indicated combinations of HA-Gnpat (A), Far1-V5 (B), Far2-V5 (C) and HA-Amacr (D) and Tysnd1 expression plasmids. (PDF) [file pgen.1003286.s005.pdf]

**Figure S7**

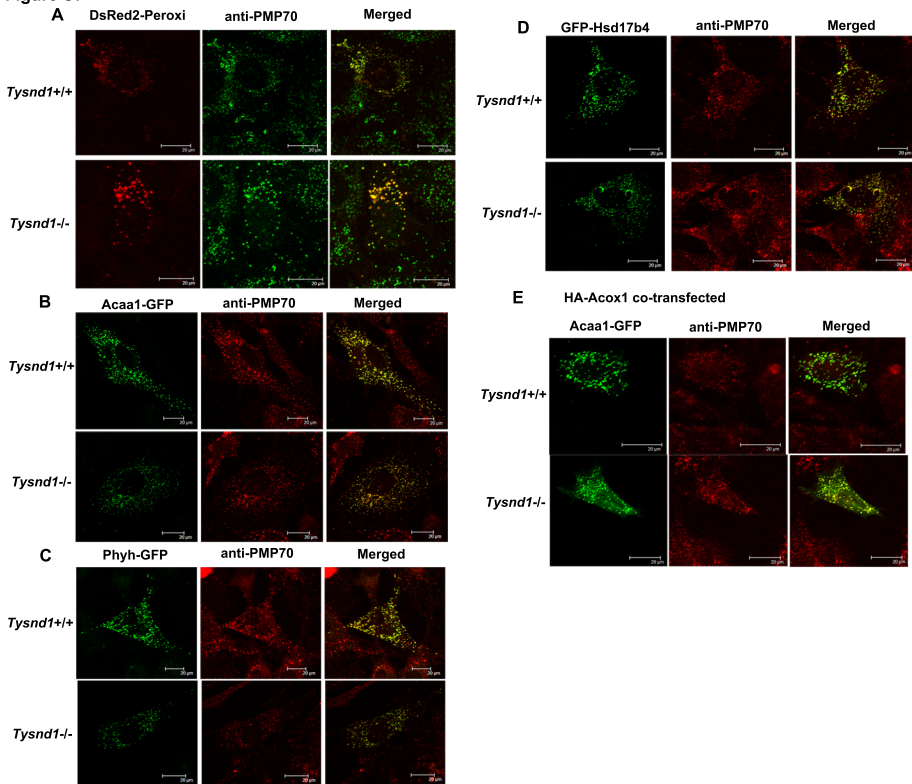

Supplement: Figure S7 — Subcellular localization of peroxisomal proteins in primary hepatocytes of Tysnd1−/− and Tysnd1+/+ mice. As a control (A) we transfected the cells with DsRed2-Peroxi and immunostained with anti-Pmp70 plus Alex Fluor 488 (green). Acaa1-GFP (B), Phyh-GFP (C), GFP-Hsd17b4 (D) and Acaa1-GFP co-transfected with HA-Acox1 (E) are shown in green and peroxisomal membrane marker Pmp70 in red after immunostaining with anti-Pmp70 and Alexa Fluor 568. (PDF) [file pgen.1003286.s007.pdf]
