# Supplementary material for: Tysnd1 Deficiency in Mice Interferes with the Peroxisomal Localization of PTS2 Enzymes, Causing Lipid Metabolic Abnormalities and Male Infertility
Source: PLoS Genet. 2013 Feb 14;9(2):e1003286. doi: 10.1371/journal.pgen.1003286 (PMC3573110; doi:10.1371/journal.pgen.1003286)
Supplement: Figure S6 — PTS2-containing proteins are imported into peroxisomes of Tysnd1 −/− primary hepatocytes. The signal intensity of GFP and DsRed2 fluorescence corresponding to the confocal-scanned images (Figure 6) was measured along the green line. Tysnd1 substrates expressed as GFP fusion proteins are shown in green peroxisome-specific DsRed2-Peroxi in red. The localization of PTS2-containing proteins Acaa1(A), Phyh(B) and Agps(C) poorly overlaps with that of DsRed2-Peroxi, while the localization of PTS1-containing proteins Acox1(D), Hsd17b4(E) and ScpX(F) coincides with DsRed2-Peroxi. (PDF) [file pgen.1003286.s006.pdf]

Figure S6

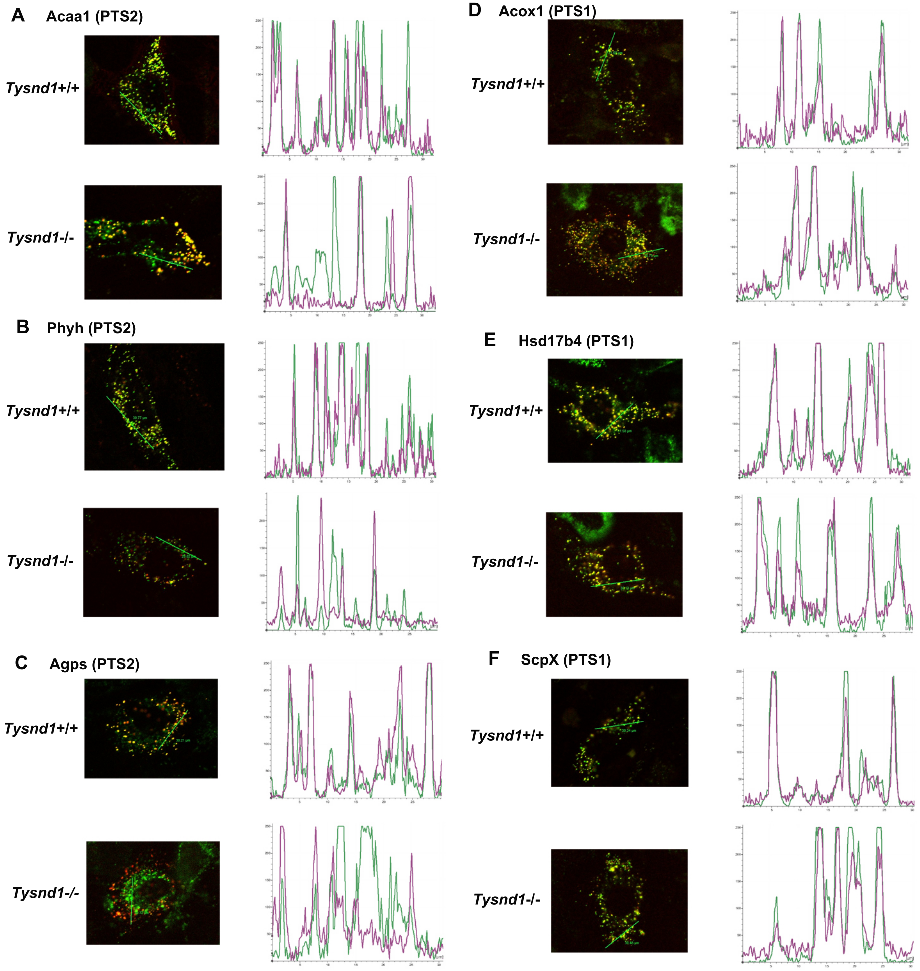
A
Acaa1 (PTS2)
Tysnd1+/+
Tysnd1-/-
B
Phyh (PTS2)
Tysnd1+/+
Tysnd1-/-
C
Agps (PTS2)
Tysnd1+/+
Tysnd1-/-
D
Acox1 (PTS1)
Tysnd1+/+
Tysnd1-/-
E
Hsd17b4 (PTS1)
Tysnd1+/+
Tysnd1-/-
F
ScpX (PTS1)
Tysnd1+/+
Tysnd1-/-
