# Supplementary material for: Tysnd1 Deficiency in Mice Interferes with the Peroxisomal Localization of PTS2 Enzymes, Causing Lipid Metabolic Abnormalities and Male Infertility
Source: PLoS Genet. 2013 Feb 14;9(2):e1003286. doi: 10.1371/journal.pgen.1003286 (PMC3573110; doi:10.1371/journal.pgen.1003286)
Supplement: Table S1 — A. Comparison of Tysnd1−/− and Tysnd1+/− pregnancies and littermate size. B. In vitro fertilization rates of Tysnd1−/− and Tysnd1+/− oocytes. (PDF) [file pgen.1003286.s008.pdf]

Table S1.

A. Comparison of *Tysnd1*$^{-/-}$ and *Tysnd1*$^{+/-}$ pregnancies and littermate size.

	***Tysnd1* Genotype**	**Pregnant /total**	**Average of littermates**
**Male**	**+/-**	**10/10**	**7**
	**-/-**	**0/18**	**0**
**Female**	**+/-**	**4/4**	**6**
	**-/-**	**6/6**	**7**

B. *In vitro* fertilization rates of *Tysnd1*$^{-/-}$ and *Tysnd1*$^{+/-}$ oocytes.

**Method**	***Tysnd1* Genotype**	**No. of oocytes used**	**No. of oocytes fertilized (%)**
**IVF Cumulus intact**	**+/-**	**433**	**347 (80.1%)***
	**-/-**	**527**	**146 (27.7%)***
**IVF Zona free**	**+/-**	**492**	**438 (89.0%)**
	**-/-**	**163**	**97 (59.5%)**
**ROSI**	**+/-**	**90**	**85 (94.4%)**
	**-/-**	**127**	**111 (87.4%)**

(A) The fertility was assessed by mating the C57BL/6J mice with *Tysnd1*$^{-/-}$, *Tysnd1*$^{+/-}$ and *Tysnd1*$^{+/+}$ mice. Eighteen female C57BL/6J mice crossed with male *Tysnd1*$^{-/-}$ mice did not become pregnant. Six female *Tysnd1*$^{-/-}$ mice that were crossed with *C57BL/6J* male mice gave birth with an average number of seven littermates. Male and female *Tysnd1*$^{+/-}$ mice were all fertile. (B) *In vitro* fertilization (IVF) under intact cumulus and zona free conditions. * p<0.01.
