# Supplementary material for: Tysnd1 Deficiency in Mice Interferes with the Peroxisomal Localization of PTS2 Enzymes, Causing Lipid Metabolic Abnormalities and Male Infertility
Source: PLoS Genet. 2013 Feb 14;9(2):e1003286. doi: 10.1371/journal.pgen.1003286 (PMC3573110; doi:10.1371/journal.pgen.1003286)
Supplement: Table S2 — Clinical blood serum biochemical analyses of Tysnd1−/− and Tysnd1+/+ mice administered with phytol. (PDF) [file pgen.1003286.s009.pdf]

Table S2. Clinical blood serum biochemical analyses of male *Tysnd1*$^{-/-}$ and *Tysnd1*$^{+/+}$ mice administered with phytol.

***Tysnd1* genotype**	**+/+**	**−/−**	**+/+**	**−/−**
**Conditions**	**0.5% CMC**	**0.5% CMC**	**phytol in CMC**	**phytol in CMC**
**LDH** [U/L]	171.0± 3.3	186.0± 6.5	174.3± 12.1	3,398.8± 476.1*
**GOT** [U/L)	39.3± 0.7	37.7± 0.7	73.8± 10.2	1,425.3± 211.1*
**GPT** [U/L]	15.9 ± 1.0	18.3 ±1.5	50.3 ±8.0	286.4 ± 48.5
**GGT** [U/L]	11.8 ± 3.0	18.1 ± 7.4	0.9 ± 0.1	3.8 ± 0.5 *
**ALP** [U/L]	305.5 ± 6.4	340.9 ± 18.1	371.9 ± 5.4	1,917.1 ± 325.7

LDH: L-lactate dehydrogenase; GOT: glutamic-oxaloacetic transaminase; GPT: glutamic-pyruvic transaminase; GGT: gamma-glutamyl transpeptidase; ALP: alkaline phosphatase; CMC: carboxyl methyl cellulose; U/L units per liter; * indicates $p<0.05$
