# Supplementary material for: Tysnd1 Deficiency in Mice Interferes with the Peroxisomal Localization of PTS2 Enzymes, Causing Lipid Metabolic Abnormalities and Male Infertility
Source: PLoS Genet. 2013 Feb 14;9(2):e1003286. doi: 10.1371/journal.pgen.1003286 (PMC3573110; doi:10.1371/journal.pgen.1003286)
Supplement: Table S3 — Polyclonal and monoclonal antibodies and their antigenic peptides. (PDF) [file pgen.1003286.s010.pdf]

Table S3. Polyclonal and monoclonal antibodies and their antigenic peptides.

Proteins	UniProt/Genbank accessions/Catalog #	Antigenic sequence	Positions
**Tysnd1**	BAB23793	SNTRDNNTGATYPHL	501-515
**Acaa1**	Q921H8, Q8VCH0	KLKPAFKOGGSTTAGNC	259-274
**Acox1**	BAA86870	TTAQQEKWMHPSQE	116-129
**ScpX**	AAH34613 ScpX (1-547); Scp2 (1-143 is identical to 405-547 of ScpX)	GTKFSDRTTPTDKH	130-143(ScpX)
**ScpX** and **Scp2**		NGKGSVLPNSDKKAD	480-494 (ScpX) 76-90 (Scp2)
**Hsd17b4**	P51660	RNQPMTPEAVRDNW	259-273
**Agps**	BC063086	KKGQVELTGKRYPLS	94-108
**Phyh**	BC029512/#12858-1-AP	purchased from Proteintech	no information
**Pex7**	BC006268/#20614-1-AP	purchased from Proteintech	N-terminal
**Pex5**	P50542/#GTX109798 recognizes short (Pex5pS) and long (Pex5pL) isoforms	purchased from GeneTex	31-311
**Abcd3** (Pmp70)	P16970/#SAB4200181 (monoclonal)	purchased from Sigma NYEFKKITEDTVEFGS	644-659
**Gapdh**	P16858/#G9545	purchased from Sigma NEYGYSNRVVDLMAYMASKE	314-333
