# Supplementary material for: Tysnd1 Deficiency in Mice Interferes with the Peroxisomal Localization of PTS2 Enzymes, Causing Lipid Metabolic Abnormalities and Male Infertility
Source: PLoS Genet. 2013 Feb 14;9(2):e1003286. doi: 10.1371/journal.pgen.1003286 (PMC3573110; doi:10.1371/journal.pgen.1003286)
Supplement: Table S4 — Primer sequences used for real time RT-PCR and constructing pcDNA3.1-V5, -HA and -GFP expression vectors. (PDF) [file pgen.1003286.s011.pdf]

Table S4. Primer sequences used for real time RT-PCR and constructing pcDNA3.1-V5, -HA and -GFP expression vectors.

Name	Sequence
**Tysnd1** (real time RT-PCR)	Forward 5'-AGCGGAGGACCTCTCTTCTC-3'<br>Reverse 5'-GTGATGGGGATGCTGAAGTT-3'
**Phyh primer for V5 vector**	Forward 5'- ACCATGGATCTTACGCGCGCCGGCGCC<br>Reverse 5'-AATGTTTATTCTTTCTCCTTTCACAAGTCGGCTTC
**Far1 primer for V5 vector**	Forward 5'-GGGCTCAGACACCATGGTTTCAATCCCAGAATACTAC<br>Reverse 5'-GGGGGATCCAATTCTTGGTCTTCTGTATCTCAT
**Far2 primer for V5 vector**	Forward 5'-GGGGGATCCCACCATGTCCATGATCGCAGCTTTCTAC<br>Reverse 5'-GGGAAGCTTCAGCTTGAACAAGGGACAAATGAA
**Gnpat primer for HA vector**	Forward 5'-GGGGGATCATGGACGTTCCTAGCTCCTCCA<br>Reverse 5'-GGGAAGCTTTTATAGTTTTGCAGTGGCTGGT
**Amacr primer for HA vector**	Forward 5'-GGGGGATCCATGGTGCTGCGTGGCGTCAGGGTTGT<br>Reverse 5'-GGGGGTACCTCAGAGATTGGCTTTTAGCTTATCAC
**Phyh primer for GFP vector**	Forward 5'- ACCATGGATCTTACGCGCGCCGGCGCC<br>Reverse 5'- TGTTTATTCTTTCTCCTTTCACAAGTCGGCTTCG
**Agps primer for GFP vector**	Forward 5'-AAGGCGGAAGCCATGGCGGAGGCGGCGGCGGG<br>Reverse 5'-TTAAAAGGTTTCTGTTTCCAAAGATGTTACTTGGG
**Acox1 primer for GFP vector**	Forward 5'- ATGAATCCCGATCTGCGCAAGGAGCG<br>Reverse 5'- CTTCAAAGCTTCGACTGCAGGGGCTTC
**Hsd17b4 primer for GFP vector**	Forward 5'- ATGGCTTCGCCGCTGAGGTTCGACG<br>Reverse 5'- CTTCAGAGCTTGGCATAGTCTTTAAGAATCATC
**ScpX primer for GFP vector**	Forward 5'- ATGCCTTCTGTCGCTTTGAAATCTCCG<br>Reverse 5'- CCTCACAGCTTAGCTTTGCCCGGCTGAA
