# Supplementary material for: Tysnd1 Deficiency in Mice Interferes with the Peroxisomal Localization of PTS2 Enzymes, Causing Lipid Metabolic Abnormalities and Male Infertility
Source: PLoS Genet. 2013 Feb 14;9(2):e1003286. doi: 10.1371/journal.pgen.1003286 (PMC3573110; doi:10.1371/journal.pgen.1003286)
Supplement: Text S1 — Supporting Methods. (PDF) [file pgen.1003286.s012.pdf]

Text S1. Supporting Methods

Determination of pristanic acid levels

Pristanic acid levels were determined from plasma samples as described before[1]. In brief, a rapid ultra-performance liquid chromatography tandem mass spectrometry (UPLC-MS/MS) method for the routine analysis of plasma pristanic acid was used. The method utilizes only 20 µl of plasma mixed with a mixture of a stable isotope-labelled internal standards mixture ($^{2}H_{3}$-Phytanic and $^{2}H_{3}$-Pristanic acids) followed by a single step of extraction with *n*-hexane and one derivatization step using 4-[2-(*N,N*-di-methylamino)ethylaminosulfonyl]-7-(2-aminoethylamino)-2,1,3-benzoxadiazole (DAABD-AE). A 5 µl sample volume was injected into UPLC-MS/MS within analytical time of 5 min.

References

1. Al-Dirbashi OY, Santa T, Rashed MS, Al-Hassnan Z, Shimozawa N, Chedrawi A, Jacob M, Al-Mokhadab M. (2008) Rapid UPLC-MS/MS method for routine analysis of plasma pristanic, phytanic, and very long chain fatty acid markers of peroxisomal disorders. J Lipid Res 49: 1855-1862.
